# Structural and catalytic characterization of *Blastochloris viridis* and *Pseudomonas aeruginosa* homospermidine synthases supports the essential role of cation–π interaction

**DOI:** 10.1107/S2059798321008937

**Published:** 2021-09-23

**Authors:** F. Helfrich, Axel J. Scheidig

**Affiliations:** a Zoological Institute, University of Kiel, Am Botanischen Garten 1–9, 24118 Kiel, Germany

**Keywords:** polyamine metabolism, Rossmann fold, NAD, putrescine, transferases, cation–π interactions, *Pseudomonas aeruginosa*, *Blastochloris viridis*, homospermidine synthases

## Abstract

Polyamines influence medically relevant processes in the opportunistic pathogen *Pseudomonas aeruginosa*, including virulence, biofilm formation and susceptibility to antibiotics. Although homospermidine synthase (HSS) is part of the polyamine metabolism in various strains of *P. aeruginosa*, neither its role nor its structure has been examined so far. The reaction mechanism of the nicotinamide adenine dinucleotide (NAD^+^)-dependent bacterial HSS has previously been characterized based on crystal structures of *Blastochloris viridis* HSS (*Bv*HSS). This study presents the crystal structure of *P. aeruginosa* HSS (*Pa*HSS) in complex with its substrate putrescine. A high structural similarity between *Pa*HSS and *Bv*HSS with conservation of the catalytically relevant residues is demonstrated, qualifying *Bv*HSS as a model for mechanistic studies of *Pa*HSS. Following this strategy, crystal structures of single-residue variants of *Bv*HSS are presented together with activity assays of *Pa*HSS, *Bv*HSS and *Bv*HSS variants. For efficient homospermidine production, acidic residues are required at the entrance to the binding pocket (‘ionic slide’) and near the active site (‘inner amino site’) to attract and bind the substrate putrescine via salt bridges. The tryptophan residue at the active site stabilizes cationic reaction components by cation–π interaction, as inferred from the interaction geometry between putrescine and the indole ring plane. Exchange of this tryptophan for other amino acids suggests a distinct catalytic requirement for an aromatic interaction partner with a highly negative electrostatic potential. These findings substantiate the structural and mechanistic knowledge on bacterial HSS, a potential target for antibiotic design.

## Abstract 2

The homospermidine synthases from *P. aeruginosa* and *B. viridis*, as well as their single-residue variants, are compared based on crystal structures and activity assays. A high structural similarity is demonstrated, suggesting the equivalent involvement of relevant residues in the reaction mechanism and catalytic dependence on cation–π interaction.

## 1. Introduction   

Polyamines are involved in various processes in nearly all organisms in the three domains of life (Michael, 2016 ▸). The composition and roles of polyamines in prokaryotes are highly diverse and species-specific (Hamana *et al.*, 2007 ▸; Hamana & Matsuzaki, 1992 ▸). *Pseudomonas aeruginosa* is a Gram-negative opportunistic pathogen that is frequently responsible for nosocomial infections (Walter *et al.*, 2018 ▸) and is known to develop multi-drug-resistant or even pan-drug-resistant strains (Horcajada *et al.*, 2019 ▸). Polyamines in *P. aeruginosa* have been demonstrated to be required for growth (Bitonti *et al.*, 1982 ▸) and to affect biofilm formation (Cardile *et al.*, 2017 ▸; Qu *et al.*, 2016 ▸; Williams *et al.*, 2010 ▸) and the susceptibility to antibiotics (Kwon & Lu, 2006*a* ▸,*b* ▸, 2007 ▸). Expression of the type III secretion system, a major virulence determinant in *P. aeruginosa*, was discovered to be influenced by the polyamine transporter SpuDEFGH (Anantharajah *et al.*, 2016 ▸; Wu *et al.*, 2012 ▸; Zhou *et al.*, 2007 ▸). The polyamine-catabolizing enzyme PauA2 was further shown to maintain the tolerance for polyamines, as disruption of this enzyme caused exogenous polyamines to exert bactericidal effects (Yao *et al.*, 2012 ▸). While these studies suggest polyamine metabolism and the associated proteins to be potential antibiotic targets, neither homospermidine synthase (HSS; EC 2.5.1.45) nor its products have been addressed in *P. aeruginosa* so far.

**Figure 1:**
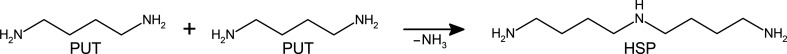
The main reaction catalyzed by HSS. Two putrescine (PUT) molecules are converted into one *sym*-homospermidine (HSP) molecule.

Bacterial HSS is mostly found in α-proteobacteria and in some β- and γ-proteobacteria, including pathogenic strains such as *Legionella pneumophila*, *Brucella* spp. and various *P. aeruginosa* strains, including PA7 and PA14 (Shaw *et al.*, 2010 ▸). It catalyzes the conversion of two molecules of putrescine (PUT; 1,4-diaminobutane) into one molecule of *sym*-homospermidine [HSP; bis(4-aminobutyl)amine] as the main reaction (Tait, 1979 ▸; Fig. 1 ▸) along with several side reactions (Ober *et al.*, 1996 ▸; Böttcher *et al.*, 1994 ▸; Supplementary Fig. S1).

Bacterial HSS is NAD^+^-dependent, active at neutral to basic pH with an optimum at pH ∼8.8, and shows increased activity in the presence of 50 m*M* potassium ion (Tait, 1979 ▸; Yamamoto *et al.*, 1993 ▸). 1,3-Diaminopropane (DAP) acts as a strongly competitive inhibitor (Tait, 1979 ▸; Yamamoto *et al.*, 1993 ▸), although it is converted with PUT into spermidine.

**Figure 2:**
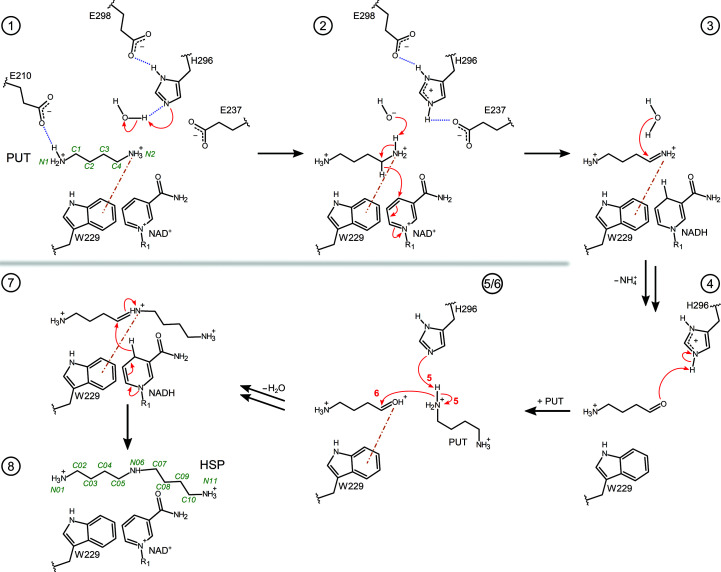
Proposed reaction steps in the conversion of PUT to HSP by bacterial HSS. The relevant residues, NAD(H), PUT, HSP and intermediates are shown as two-dimensional structure representations. Hydrogen bonds are depicted as blue dotted lines, delocalized electrons as dashed lines, cation–π interactions as orange dashed–dotted lines and electron transfers as red arrows. Atom numbering for PUT and HSP is given in green. For simplicity, steps 5 and 6 are shown as a combined depiction with correspondingly labeled electron transfers. A more detailed sequence of the reaction steps has been described previously (Krossa *et al.*, 2016 ▸) and additional intervening reaction steps are proposed in Supplementary Fig. S2.

A profound structural characterization of the bacterial HSS from *Blastochloris viridis* (*Bv*HSS) has previously been reported (Krossa *et al.*, 2016 ▸). *B. viridis* is a Gram-negative, photoheterotrophic α-proteobacterium with a simple photosynthetic system and thus is a commonly studied model organism for plant biochemistry (Konorty *et al.*, 2009 ▸). Crystal structures of wild-type *Bv*HSS and its single-residue variants in complex with various polyamines have been presented together with qualitative activity assays (Krossa *et al.*, 2016 ▸). Based on these findings, the relevant residues involved in catalysis were identified and a detailed reaction mechanism was proposed. *Bv*HSS forms a dimer, with each subunit containing a boot-shaped substrate-binding pocket between an NAD(P)-binding Rossmann-like domain and an HSS-like domain. The cofactor NAD^+^ is bound as a prosthetic group in the binding pocket with its nicotinamide ring being part of the active site. An ‘ionic slide’ (residues Asp94 and Glu117) was proposed to lead positively charged amine substrates from the entrance to the binding pocket into the active site. The entrance tunnel is lined by a so-called ‘track-and-trace’ loop (residues 114–130). Krossa and coworkers identified three binding sites for the amino groups of reaction components within the substrate-binding pocket (Krossa *et al.*, 2016 ▸). Assuming the amino groups of the reaction components to be protonated at neutral to slightly basic pH, the ‘innermost’ N atoms of PUT (N1) and of HSP (N01) (for numbering, see Fig. 2 ▸) mainly bind via a salt bridge to Glu210 and, in addition, via a hydrogen bond to Asn162 at the ‘inner amino site’. At the ‘outer amino site’, the ‘outer’ amino group of HSP (N11) forms a salt bridge with Glu237 and a hydrogen bond to the O2D atom of NAD^+^. The N atoms PUT N2 and HSP N06 are located at the ‘center amino site’ between Asn162 and His296 as well as between the ring planes of the Trp229 indole ring and the nicotinamide ring. The residues constituting the ‘center amino site’ also form the active site by surrounding the C atoms PUT C4 and HSP C05. The nicotinamide ring of NAD^+^ is assumed to serve as a hydride acceptor and donor (Böttcher *et al.*, 1994 ▸), while His296 acts as a strong base by being arranged in a triad with the neighboring residues Glu237 and Glu298. Based on the interaction geometry between the Trp229 indole plane and the bound PUT and HSP molecules (PDB entry 4tvb; Krossa *et al.*, 2016 ▸), the aromatic indole ring was suggested to stabilize positively charged amino groups and transiently charged C atoms of reaction components and intermediates by cation–π inter­action during catalysis (Krossa *et al.*, 2016 ▸). Cation–π interactions are noncovalent inter­actions (Dougherty, 1996 ▸; Sunner *et al.*, 1981 ▸) and can approximately be explained by electrostatic models (Mecozzi *et al.*, 1996*a* ▸,*b* ▸) as the attraction between the positive charge of a cation and the negative partial charge of the quadrupole moment of a π system. Binding energies are in the range of those of hydrogen bonds and salt bridges in aqueous solution (Pletneva *et al.*, 2001 ▸). The key steps of the previously proposed reaction mechanism (Krossa *et al.*, 2016 ▸) are described in the following, assuming the amino groups of the reaction components to be protonated (Fig. 2 ▸, steps 1–8).

Initially, His296, as a strong base, probably generates a hydroxide anion, which in turn deprotonates the cation–π-stabilized PUT amino group at the ‘center amino site’ (steps 1–2). A hydride transfer from PUT C4 to NAD^+^ C4N yields a protonated imine (steps 2–3). The positive charge of the imine might be transiently stabilized at C4 by a geometrically more favorable cation–π interaction with C4 than with N2 (Supplementary Fig. S2, step 4). This would further facilitate a nucleophilic attack by a water molecule, resulting in the formation of 4-aminobutanal by deamination (steps 3–4). After protonation of the aldehyde O atom by His296 (steps 4–5), a positive charge might again be transiently stabilized at the C4 atom (PUT nomenclature) of the aldehyde by cation–π interaction to facilitate another nucleophilic attack (Supplementary Fig. S2, step 9). The amino group of a second PUT molecule is deprotonated by His296 (step 5) and performs a nucleophilic attack at the C4 atom (step 6), generating a protonated Schiff base (step 7). Hydride transfer from NADH ultimately results in the production of HSP (steps 7–8), which might accept a proton at its center amino group from the still protonated His296 before leaving the active site. Apart from the stabilization of transient carbocations, the Trp229 indole ring was also proposed to be involved in (geometrically less favored) cation–π interactions with the positively charged nitrogen moieties of intermediates such as the protonated imine (step 3) and the protonated Schiff base (step 7). This reaction mechanism is supported by four inactive variants affecting the Glu–His–Glu triad (E237Q, H296S and E298Q) and the ‘center amino site’ (N162D) (Krossa *et al.*, 2016 ▸). However, the roles of other residues, including the ‘ionic slide’ (Asp94 and Glu117), Glu210 at the ‘inner amino site’ and Trp229 as a potential cation–π interaction partner, were not experimentally evaluated. In addition, the question arises as to whether these findings can be transferred to bacterial HSS from medically more relevant species such as *P. aeruginosa.*

To address these questions, we present the crystal structure of *P. aeruginosa* HSS (*Pa*HSS; PDB entry 6y87) and confirm a high structural similarity between *Pa*HSS and *Bv*HSS, including the location of the catalytically relevant residues. We chose *Bv*HSS as a model for further mechanistic characterization of *Pa*HSS and of bacterial HSS in general because (i) a high sequence conservation of the catalytically relevant residues (*Bv*HSS numbering: Glu117, Asn162, Glu210, Trp229, Glu237, His296 and Glu298) in bacterial HSS orthologs has previously been reported (Shaw *et al.*, 2010 ▸), (ii) *Pa*HSS and *Bv*HSS consistently exhibit high structural similarity, as presented in this study, and (iii) *Bv*HSS crystals provide superior X-ray diffraction data compared with *Pa*HSS crystals. To substantiate the previously proposed roles of catalytically relevant residues, we have produced additional single-residue variants of *Bv*HSS for characterization by X-ray crystallo­graphy and by quantitative activity assays. Our results confirm the important roles of the residues forming the ‘ionic slide’ and the ‘inner amino site’ and support the requirement for the active-site tryptophan as a potent cation–π interaction partner. In the context of polyamine metabolism in *P. aeruginosa* being a potential target for antibiotic design, this study might provide possible approaches to interfere with HSP production in *P. aeruginosa* and other bacteria.

## 2. Materials and methods   

### 2.1. Materials   

Primers were obtained from Eurofins Genomics (Ebersberg, Germany). DNA purification kits were purchased from Macherey-Nagel (Düren, Germany). Pre-packed chromatography columns (HisTrap HP, HiTrap Q HP, GSTrap HP and HiTrap Desalting) for preparative protein purification were obtained from GE Healthcare (Chicago, Illinois, USA). Other reagents, unless stated otherwise, were reagent grade or better and were purchased from Carl Roth (Karlsruhe, Germany) or Sigma–Aldrich (Darmstadt, Germany).

### 2.2. Cloning of the *Pa*HSS expression plasmid   

The amino-acid sequence of *Pa*HSS (UniProtKB Q6X2Y9) was back-translated into an *Escherichia coli* K12 codon-optimized DNA sequence and extended by terminal 5′-CC and TAATAACTCGAG-3′ DNA sequences (Supplementary Fig. S3). The resulting DNA sequence was synthesized by General Biosystems Inc. (Durham, North Carolina, USA) and was provided as an insert in a pUC57 plasmid. This plasmid was transformed into *E. coli* XL1-Blue cells for plasmid production. The *Pa*HSS-coding DNA sequence was transferred from the pUC57 plasmid into a pETM-14 plasmid (EMBL, Heidelberg, Germany) via the NcoI/XhoI restriction sites on both plasmids (endonucleases from New England Biolabs GmbH, Frankfurt am Main, Germany) and ligated with T4 DNA Ligase (Thermo Fisher Scientific, Waltham, Massachusetts, USA), yielding the *Pa*HSS pETM-14 plasmid. The plasmid codes for an N-terminal 6×His tag followed by a human rhinovirus 3C (HRV3C) protease cleavage site fused to the *Pa*HSS sequence. The *Pa*HSS pETM-14 plasmid was transformed into *E. coli* XL1-Blue cells for plasmid amplification and the correct sequence of the *Pa*HSS insert was confirmed by DNA sequencing (Eurofins Genomics). For protein expression, *Pa*HSS pETM-14 was transformed into *E. coli* BL21(DE3) Gold cells.

### 2.3. Cloning of expression plasmids for *Bv*HSS variants   

The expression plasmid *Bv*HSS pETM-14 coding for wild-type *Bv*HSS (UniProtKB O32323) was previously generated (Krossa *et al.*, 2016 ▸). Expression of the plasmid yields wild-type *Bv*HSS with an N-terminal 6×His tag followed by an HRV3C protease cleavage site. For the generation of single-residue *Bv*HSS variants, the QuikChange site-directed mutagenesis protocol from Agilent was followed using Phusion high-fidelity polymerase (Thermo Fisher Scientific), *Bv*HSS pETM-14 as the template and variant-specific primer pairs (Supplementary Table S1). After PCR, the template plasmid was digested with the endonuclease DpnI (Thermo Fisher Scientific), followed by the transformation of PCR products into *E. coli* XL1-Blue cells for clone screening and confirmation of the correct construct sequence by DNA sequencing. For protein expression, plasmids were transformed into *E. coli* BL21(DE3) Gold cells.

### 2.4. Protein expression and purification   

Protein expression of wild-type *Bv*HSS and *Bv*HSS variants was performed as described previously (Krossa *et al.*, 2016 ▸). The expression of *Pa*HSS was similarly conducted with two modifications of the *Bv*HSS expression protocol to increase the yield of soluble *Pa*HSS: the induction of expression was performed with 0.1 m*M* isopropyl β-d-1-thiogalactopyranoside (instead of 1 m*M*) and expression was continued for 20 h after induction (instead of 4 h).

For the purification of *Pa*HSS, the cells were resuspended 1:10(*w*:*v*) in basis buffer [50 m*M* Tris–HCl pH 7.4, 25 m*M* KCl, 2 m*M* dithiothreitol (DTT)] supplied with 0.3 *M* NaCl, 25 m*M* imidazole, 2 m*M* NAD^+^ and 1 m*M* phenylmethanesulfonyl fluoride. The cells were lysed by one passage through an Emulsiflex C3 homogenizer (Avestin, Mannheim, Germany) at pulses of approximately 1200 bar. After centrifugation (45 min, 75 600*g*, 283 K), *Pa*HSS was purified from the clarified supernatant via chromatographic purification steps using an ÄKTA FPLC system (GE Healthcare) equipped with UV and conductivity detectors. The supernatant was first loaded onto an equilibrated [15 column volumes (CV) basis buffer] 5 ml HisTrap HP column. The column was then washed with 5 CV basis buffer supplied with 25 m*M* imidazole and elution was subsequently performed at ∼0.2 *M* imidazole by linearly increasing (25 m*M* imidazole per CV) the concentration of imidazole in basis buffer to 0.5 *M*. The buffer of the eluted protein was exchanged for fresh basis buffer supplied with 2 m*M* NAD^+^ using 5 ml HiTrap Desalting columns. The N-terminal 6×His tag was removed by incubating the *Pa*HSS with 1:100(*w*:*w*) glutathione *S*-transferase (GST)-tagged HRV3C protease overnight at 277 K, followed by passage of the sample through connected 5 ml HisTrap HP and GSTrap HP columns in basis buffer. The flowthrough was loaded onto two connected 1 ml HiTrap Q HP columns equilibrated with basis buffer. *Pa*HSS was eluted between ∼18 and 23 mS cm^−1^ by linearly increasing (20 m*M* NaCl per CV) the NaCl concentration in basis buffer to 0.3 *M* NaCl. The eluted sample was supplied with fresh basis buffer using HiTrap Desalting columns and was concentrated to ∼5 mg ml^−1^ using 10 kDa Amicon Ultra-15 centrifugal filter units (Merck Millipore, Darmstadt, Germany). After the addition of 2 m*M* NAD^+^, the *Pa*HSS sample was flash-frozen in liquid nitrogen and stored at 193 K.

Purification of wild-type *Bv*HSS and *Bv*HSS variants was performed as described previously (Krossa *et al.*, 2016 ▸), but the standard buffer used in this study was composed of 50 m*M* bis-Tris propane–HCl pH 9, 25 m*M* KCl, 2 m*M* DTT.

Protein concentrations were estimated according to the Lambert–Beer law by measurement of the absorbance at 280 nm. The respective extinction coefficients were calculated with the *ProtParam* tool (Gasteiger *et al.*, 2005 ▸). Reductive SDS–PAGE analysis revealed a purity of >99% for wild-type *Bv*HSS and the *Bv*HSS variants and >95% for *Pa*HSS. The oligomerization states of the proteins were determined by SEC-MALS as described in Supplementary Section S1.

### 2.5. HSS activity assay   

To quantify the conversion of PUT to HSP, 10 m*M* PUT dihydrochloride was mixed into three prewarmed reaction pools (independent triplicate measurements) consisting of 3.8 µ*M* protein, 50 m*M* potassium phosphate pH 7.4 and 0.2 m*M* NAD^+^ at 310 or 298 K. At different time points, 100 µl from the reaction pools was transferred into 50 µl 100%(*w*/*v*) trichloroacetic acid to stop the reaction. Precipitated protein was removed by centrifugation (18 000*g* for 10 min) and 1 µl supernatant was neutralized in 589 µl 0.2 *M* sodium borate–NaOH buffer pH 8.5. 25 µl of the neutralized sample was mixed with 10 µl 10 m*M* 6-aminoquinolyl-*N*-hydroxy­succinimidyl carbamate (Synchem UG & Co. KG, Felsberg, Germany) in dry acetonitrile to label the polyamines. After incubation for 5 min at room temperature in the dark, the sample was diluted with 35 µl 0.2 *M* sodium borate–NaOH buffer pH 8.5, and 50 µl was loaded onto a ProntoSIL HyperSorb ODS (C18) column (4.6 × 250 mm, 120 Å pore size, 5 µm particle size; Bischoff Chromatography, Leonberg, Germany) connected to the HPLC system described in Section S1. The mobile phase was a gradient of 25 m*M* tri­ethylamine (pH 4.8, adjusted with acetic acid), 80%(*v*/*v*) acetonitrile and methanol (Table 1 ▸) as described previously (Weiss *et al.*, 1997 ▸).

The flow rate was 1.3 ml min^−1^ at 306 K and fluorescence was measured at λ_ex_ = 248 nm and λ_em_ = 398 nm at a photomultiplier gain of 11. Signal peaks were integrated for quantification using the *ChemStation* software version B.01.03 (Agilent). To confirm the retention times of the respective labeled compounds and to convert fluorescence peak areas to compound quantities, different amounts of PUT and HSP (Toronto Research Chemicals Inc., Ontario, Canada) were directly labeled and separated on the column (Supplementary Figs. 4*a* and 4*b* ) to determine a standard curve (Supplementary Fig. 4*c* ). The mean concentration of the produced HSP was plotted against the reaction time using *GraphPad Prism* version 5.00 (Supplementary Figs. 4*d* and 4*e* ). The initial velocities (and their standard deviations) were calculated from the linear fit to all data points (not only the mean) of the initial, linear part of each progression curve, considering at least three time points.

### 2.6. Crystallization and data collection   

Crystallization was performed by the hanging-drop vapor-diffusion technique at 291 K. *Pa*HSS was crystallized in drops consisting of 1 µl protein solution (∼3.3 mg ml^−1^ *Pa*HSS, 33 m*M* Tris–HCl pH 7.4, 17 m*M* KCl, 1.3 m*M* DTT, 1.3 m*M* NAD^+^, 0.2 *M* agmatine sulfate) and 1 µl reservoir solution [0.1 *M* HEPES–NaOH pH 7.5, 18–23%(*w*/*v*) poly(acrylic acid sodium salt) average molecular weight 5100 (Sigma–Aldrich), 10 m*M* adenosine triphosphate] by equilibration against 0.5 ml reservoir solution. The addition of agmatine sulfate was found to significantly improve the growth and diffraction quality of the crystals. Crystals appeared after ∼1–3 days and were disintegrated by vortexing with glass beads in reservoir solution. Crystal nuclei were streak-seeded into hanging drops of a fresh crystallization setup prepared as described above. Homogeneous, trigonal prism-shaped crystals grew within several days in a narrow concentration range of poly(acrylic acid sodium salt) and were briefly soaked with a mixture of 20%(*v*/*v*) glycerol and 80%(*v*/*v*) reservoir solution before vitrification in liquid nitrogen.

*Bv*HSS variants were crystallized as described by Krossa *et al.* (2016 ▸). The hanging drop consisted of a mixture of 1 µl protein solution (∼3.3 mg ml^−1^ HSS, 33 m*M* bis-Tris propane–HCl pH 9, 17 m*M* KCl, 1.3 m*M* DTT, 1.3 m*M* NAD^+^, 0.2 *M* agmatine sulfate) and 1 µl reservoir solution [0.1 *M* sodium acetate pH 4.6–4.8, 150 m*M* ammonium acetate, 22–26%(*w*/*v*) polyethylene glycol (PEG) 10 000, 150–300 m*M* 3-(1-pyridino)-1-propane sulfonate] equilibrated against 0.5 ml reservoir solution. The PEG 10 000 in the crystallization solution simultaneously served as a cryoprotectant. The addition of agmatine sulfate improved the growth and diffraction quality of the crystals. Crystals became visible after ∼3 days in needle clusters. Single needles were manually isolated from the clusters and either directly vitrified in liquid nitrogen (variants E117Q, PDB entry 6s6g; E210A, PDB entry 6s49; E210Q, PDB entry 6s3x) or soaked for 5 min in a mixture of 0.2 *M* PUT dihydrochloride (final soaking concentration) in 2/3 reservoir solution prior to vitrification (variants W229A, PDB entry 6s72; W229E, PDB entry 6sep; W229F, PDB entry 6s4d). Longer soaking times (>15 min) generally decreased the integrity and diffraction quality of the crystals.

Diffraction data were collected at 100 K on beamlines P13 (detector, Dectris PILATUS3 6M; wavelength, 1.0332 Å; E117Q, E210A, W229E and W229A *Bv*HSS variants) and P14 (detector, Dectris EIGER X 16M; wavelength, 0.9789 Å for the E210Q *Bv*HSS variant and 0.9763 Å for the W229F *Bv*HSS variant and *Pa*HSS) at the PETRA III synchrotron at DESY (EMBL, Hamburg, Germany) in oscillation increments of 0.1° per image.

### 2.7. Structure determination and representation   

Indexing and integration of diffraction data was performed with *XDS* (Kabsch, 2010 ▸). Using the *CCP*4 program suite (Evans, 2011 ▸; Potterton *et al.*, 2003 ▸), the space group was estimated with *POINTLESS*. Scaling, data reduction and assignment of free *R* flags to 5% of the reflections were performed with the *CCP*4 program *SCALA*. Molecular replacement and automated refinement were performed using *Phenix* (Liebschner *et al.*, 2019 ▸). Inspection of molecular-replacement results and manual model building between automated refinement cycles was performed by *Coot* (Emsley *et al.*, 2010 ▸). For automated refinement by *Phenix*, the included refinement parameters were atomic positions (Cartesian and real space), individual *B* factors and occupancies. The ‘maximum-likelihood’ target function was applied and weight optimization was enabled for the X-ray target function and geometry restraints as well as *B*-factor restraints. Directly after molecular replacement, rigid-body refinement and Cartesian simulated annealing was included in the refinement. At later stages in the refinement, automatic addition/updating of H atoms and water molecules was performed. All structures presented in this study were solved by molecular replacement using the *Phaser* maximum-likelihood molecular-replacement tool implemented in *Phenix*.

For the *Bv*HSS variants, the wild-type *Bv*HSS structure (PDB entry 4plp; Krossa *et al.*, 2016 ▸), excluding the NAD^+^ molecules and any other ligands, served as a search model. After molecular replacement, the mutated residue of each variant was accordingly exchanged and the NAD^+^ molecules were fitted into the binding pockets. After refinement including Cartesian simulated annealing, the model was iteratively optimized by manual model building and automated refinement.

For the *Pa*HSS structure (PDB entry 6y87), a *Pa*HSS homology model without the NAD^+^ molecule or other ligands was calculated using the standard settings of the *PRIMO* server (Hatherley *et al.*, 2016 ▸) and was used as a search model. For the homology model, the wild-type *Bv*HSS model (PDB entry 4plp; Krossa *et al.*, 2016 ▸) and the *Pa*HSS amino-acid sequence (UniProtKB Q6X2Y9) served as input. Six molecules of *Pa*HSS were estimated to fit into the asymmetric unit according to the Matthews coefficient calculator software within the *CCP*4 suite (Table 2 ▸). In the *Phaser* maximum-likelihood molecular-replacement tool, automated molecular replacement was performed with six *Pa*HSS homology models as search models per asymmetric unit, enabling the option to try both enantiomeric space groups. After the correct placement of six *Pa*HSS search models within the asymmetric unit, NAD^+^ molecules were fitted into each active site and refinement including Cartesian simulated annealing was performed. The models were further improved by iterative manual and automated refinement.

Structural images were prepared with *PyMOL* (Schrödinger). Omit maps were calculated both with the composite omit map tool and the polder map tool implemented in *Phenix* (Liebschner *et al.*, 2017 ▸). Using the composite omit map tool, only one specified part of the model was omitted, followed by refinement including Cartesian simulated annealing to remove model bias. Bulk solvent was allowed to occupy omit regions in the refinement step. For PUT composite omit maps, only the PUT molecule was omitted. For composite omit maps targeting the exchanged residue in each *Bv*HSS variant, the mutated residue together with ten N-terminally and ten C-terminally adjacent residues were omitted. Polder maps were calculated by omitting the PUT molecules and exclusion of the bulk solvent within a distance of 5 Å around the ligands.

Superimposition of *Pa*HSS molecule *A* onto the other *Pa*HSS molecules (PDB entry 6y87; Supplementary Table S2) as well as superimposition of wild-type *Bv*HSS onto *Bv*HSS variants (Supplementary Table S3) was performed with *PyMOL* using the *super* algorithm over five cycles. Therefore, only protein atoms and NAD^+^ atoms without hydrogens and only alternative conformations *A* were considered. Root-mean-square distance (r.m.s.d.) values were subsequently calculated with the command rms_cur. For the generation of a structure-based sequence alignment of *Bv*HSS (PDB entry 4tvb chain *B*) with *Pa*HSS (PDB entry 6y87 chain *A*; Supplementary Figs. S5 and S6) and of *Pa*HSS (PDB entry 6y87 chain *A*) with *L. pneumophila* HSS (*Lp*HSS; PDB entry 2ph5; Supplementary Fig. S7), the *MatchMaker* tool of *UCSF Chimera* 1.14 (Pettersen *et al.*, 2004 ▸) was used. The initial sequence alignment was based on the Needleman–Wunsch algorithm, the BLOSUM62 substitution matrix and secondary-structure scoring. The subsequent superimposition of structures included iterative pruning of C^α^-atom pairs which exceeded a distance of 2 Å. The resulting structure-based sequence alignment underlies a C^α^-based residue–residue distance cutoff of 5 Å.

For the depiction of solvent-accessible surfaces of binding pockets, pockets were filled with dummy atoms (surface probe sphere radius, 4 Å; dummy-atom radius, 1.4 Å; grid spacing, 0.2 Å) using *HOLLOW* 1.1 (Ho & Gruswitz, 2008 ▸). After the addition of water molecules present in the respective structures, the surface of these dummy atoms was depicted using *PyMOL*.

## 3. Results and discussion   

### 3.1. Organization of six highly similar *Pa*HSS molecules in the asymmetric unit   

The *Pa*HSS structure was solved in space group *P*3_2_12 using the *Phaser* tool implemented in *Phenix* and allowing both enantiomorphic space groups as possible solutions. Six *Pa*HSS molecules were modeled into the asymmetric unit and refined to a maximum resolution of 2.42 Å (Table 2 ▸). All *Pa*HSS residues of molecule *A* could be modeled into the electron-density distribution, including an N-terminal residue belonging to the remains of the cleaved affinity tag (residue 0). Due to the local absence of interpretable electron density, a few residues belonging to the N-terminal loop and to a loop near the C-terminus were not modeled in the other *Pa*HSS molecules (the missing residues were 1–3 in *Pa*HSS molecule *B*, 1–5 and 449–458 in molecule *C*, 1–4 and 449–457 in molecule *D*, 1–3 and 451–456 in molecule *E* and 1–3 and 450–457 in molecule *F*). These loops are solvent-exposed on the protein surface without influence on the binding pocket. All six *Pa*HSS molecules contain an NAD^+^ molecule in their binding pockets, and molecules *A*, *C* and *E* additionally contain a PUT molecule in the active site. The degree of structural similarity between the *Pa*HSS molecules was examined by superimposition of molecule *A* onto the other molecules. The resulting r.m.s.d. values considering all atoms (not only C^α^) were between ∼0.73 and ∼0.83 Å (Supplementary Table S2), demonstrating a high structural similarity. Determination of crystal contacts between the individual protein molecules (for PDB entry 6y87) was performed using the *PDBsum* web server (Laskowski *et al.*, 2018 ▸). In addition to various smaller to moderate contact interface areas between the molecules, larger interface areas were detected between the pairwise-associated molecules *A* and *B* (1381–1401 Å^2^), *C* and *D* (1328–1332 Å^2^), and *E* and *F* (1379–1370 Å^2^). Although *Pa*HSS was shown to be a monomer in solution by SEC-MALS (the theoretical molecular weight of the monomer and the estimated molecular weight according to HPLC-MALS were 52 kDa; data not shown), these interface areas indicate the formation of three crystalline dimers by the six protein molecules within the asymmetric unit. The monomeric state of *Pa*HSS in solution is also supported by the shape complementarity (Sc) values of the crystalline dimers (Sc values of 0.607, 0.654 and 0.612 for *A*–*B*, *C*–*D* and *E*–*F*, respectively), which are below the generally reported Sc values of 0.7–0.76 for protein oligomers (Lawrence & Colman, 1993 ▸).

### 3.2. Overall structure and binding pocket of *Pa*HSS   

**Figure 3:**
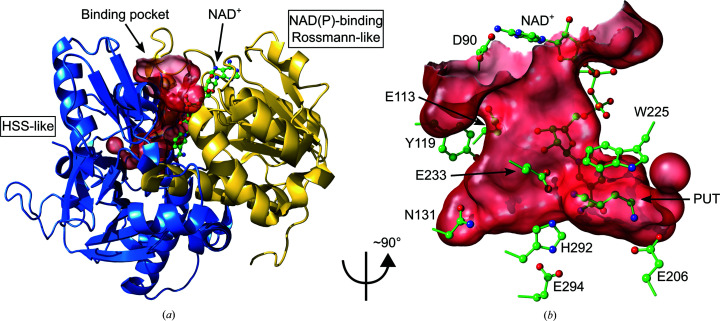
Structure and binding pocket of *Pa*HSS. (*a*) Cartoon representation of *Pa*HSS (PDB entry 6y87 chain *A*) with the NAD(P)-binding Rossmann-like domain colored yellow (residues 1–155 and 389–419) and the HSS-like domain in blue (residues 156–388 and 420–469). The solvent-accessible surface of the binding pocket is depicted in transparent red with the entrance pointing upwards. The NAD^+^ molecule lining the surface of the pocket is shown in ball-and-stick representation. (*b*) The solvent-accessible surface of the binding pocket was rotated by an angle of ∼90° around the *y* axis in relation to (*a*). Selected side chains lining the pocket surface, the NAD^+^ molecule and the PUT molecule within the active site are depicted in ball-and-stick representation.

*Pa*HSS is composed of an NAD(P)-binding Rossmann-like domain (residues 1–155 and 389–419) and an HSS-like domain (residues 156–388 and 420–469), as confirmed by the CATH database (Knudsen & Wiuf, 2010 ▸) and as previously observed for the *Bv*HSS structure (Krossa *et al.*, 2016 ▸). The binding pocket is formed between the domains and accommodates an NAD^+^ molecule (Fig. 3 ▸ *a*).

The same residues as were previously discussed to be involved in catalysis by *Bv*HSS (Krossa *et al.*, 2016 ▸) are present in the binding pocket of *Pa*HSS with the sequence numbering shifted by −4 (Fig. 3 ▸ *b*). Transferring the proposed reaction mechanism of *Bv*HSS to *Pa*HSS, the following roles could be assigned to these *Pa*HSS residues. Asp90 and Glu113 form the ‘ionic slide’ at the entrance tunnel of *Pa*HSS. Tyr119 borders the entrance tunnel and is part of the so-called ‘track-and-trace’ loop (*Pa*HSS residues 110–124). The equivalent *Bv*HSS residue Tyr123 was assumed to regulate access to the binding pocket by modifying the pore dimensions (Krossa *et al.*, 2016 ▸). The NAD^+^ molecule in *Pa*HSS lines the entrance tunnel with its pyrophosphate moiety, contributes to the ‘outer amino site’ with its O2D O atom and inserts its nicotinamide moiety into the active site. The nicotinamide ring plane, the indole ring plane of Trp225 and His292 surround the ‘center amino site’ and the active site. His292 thereby forms a triad with Glu233 and Glu294. The PUT molecule is located with its ‘outer’ amino group and C4 atom at the ‘center amino site’/active site and with its ‘inner’ amino group near Glu206 at the ‘inner amino site’. As in *Bv*HSS, *Pa*HSS forms a side pocket at the bottom of the binding pocket, which is confined by Asn131, among others.

### 3.3. PUT molecules and a potential NAD adduct in the active sites of *Pa*HSS   

**Figure 4:**
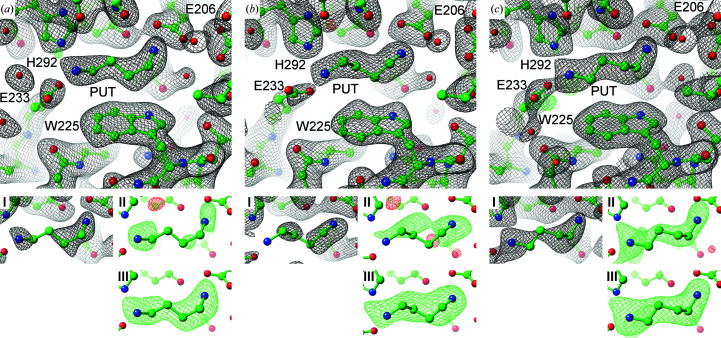
Active sites of three *Pa*HSS molecules with bound PUT molecules. Atomic models of *Pa*HSS molecules [PDB entry 6y87 chain *A* (*a*), chain *C* (*b*) and chain *E* (*c*)] are depicted in ball-and-stick representation with electron-density maps as mesh. The large images show the 2*mF*_o_ − *DF*_c_ maps (black, 1σ) and *mF*_o_ − *DF*_c_ maps (green/red, ±4σ) of the complete models. Models lacking the PUT molecules were used to calculate (I) 2*mF*_o_ − *DF*_c_ composite omit maps including simulated annealing (black, 1σ), (II) *mF*_o_ − *DF*_c_ composite omit maps including simulated annealing (green/red, ±3σ) and (III) *mF*_o_ − *DF*_c_ polder maps (green/red, ±5σ).

The electron-density distribution of *Pa*HSS molecules *A*, *C* and *E* revealed the binding of a PUT molecule in each active site (Fig. 4 ▸). PUT was not added at any stage of purification or crystallization. We assume it to originate from the expression host and to be co-purified within the active site of *Pa*HSS. The possibilities of a contaminated agmatine solution or the catalytic conversion of agmatine by *Pa*HSS was ruled out by separation of the utilized agmatine solution by reversed-phase HPLC before and after incubation with *Pa*HSS. Neither contamination nor conversion of agmatine were detected.

**Figure 5:**
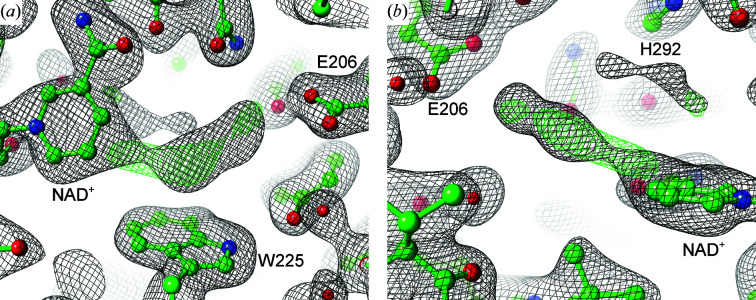
Potential NAD adduct in the active site of *Pa*HSS molecule *B*. The atomic model of the *Pa*HSS molecule (PDB entry 6y87 chain *B*) is depicted in ball-and-stick representation in two orientations in (*a*) and (*b*). The 2*mF*_o_ − *DF*_c_ map (black, 1σ) and the *mF*_o_ − *DF*_c_ map (green/red, ±4σ) are shown as mesh. The central, uninterpreted electron-density distribution could originate from a substitution at the nicotinamide ring.

Both the architecture of the active sites and the location of the PUT molecules are very similar in all three *Pa*HSS molecules. The ‘inner’ and ‘outer’ amino groups of the PUT molecules form salt bridges with the carboxyl O atoms of Glu206 and Glu233, respectively, with distances of between 2.8 and 3.2 Å. The cation–π interaction with the Trp225 indole ring is described later (Fig. 7). The conformation of the PUT carbon chains varies slightly between the respective *Pa*HSS molecules. The exact tracing of the carbon chains might be complicated by their assumed flexibility and the moderate resolution of the structure. Omit maps and polder maps were calculated to evaluate the placement of the PUT molecules in the active sites (I, II and III in Fig. 4 ▸). Inspecting these omit maps, the PUT molecule in *Pa*HSS molecule *E* was most distinctly supported by the diffraction data. The electron-density distribution at the active site of *Pa*HSS molecule *B* could not be unambiguously interpreted (Fig. 5 ▸).

The electron density contouring the bond between the nicotinamide C4N and C5N atoms clearly merges into a longitudinal, uninterpreted density, which extends to the ‘inner amino site’ near Glu206. One explanation for this observation could be radiation damage caused by the X-ray diffraction experiment. This might have turned the nicotinamide ring into a radical, which in turn resulted in adduct formation between the nicotinamide ring and another molecule. Another potential explanation for this observation could be a covalent nicotinamide adduct occurring as an intermediate in the reaction mechanism, although additional experiments will be needed to confirm such an adduct. In the wild-type *Bv*HSS structure (PDB entry 4tvb), close proximity of a PUT molecule and an HSP molecule to the nicotinamide ring was similarly observed and was suggested to reflect partial, transient hydride transfers between the respective molecules (Krossa *et al.*, 2016 ▸). These observations might instead be caused by covalent NAD adducts occurring as intermediates in the bacterial HSS. In *Bv*HSS (PDB entry 4tvb), the geometry of the potential bonds between (i) the PUT C4 and HSP C05 C atoms and (ii) the nicotinamide ring C4N atom would thereby satisfy an *sp* ^3^ hybridization of C4N. In the *Pa*HSS molecule *B* presented here, the uninterpreted density contacts the nicotinamide ring in a rather side-on geometry, which perfectly satisfies neither *sp* ^2^ nor *sp* ^3^ hybridization of C4N in the context of an adduct structure. Also, the uninterpreted density could only accommodate a smaller intermediate such as an aminobutyl moiety and not a PUT or HSP molecule. Not knowing the definite composition of the potential adduct in *Pa*HSS and relying on moderate resolution, the presence and identity of such an adduct remains to be determined by additional experiments. In line with our assumption, NAD adducts have previously been described to be formed in various enzymes between the nicotinamide ring C2N or C4N atoms and both inhibitor compounds (Jacques *et al.*, 2003 ▸; Rubach & Plapp, 2003 ▸; Benach *et al.*, 1999 ▸; Becker & Roberts, 1984 ▸) and reaction components (Vögeli *et al.*, 2018 ▸; Rosenthal *et al.*, 2014 ▸, 2015 ▸; Choe *et al.*, 2003 ▸). NAD(P) adduct formation was thereby proposed to represent an intermediate step in the mechanism of enzymatic hydride transfer (Vögeli *et al.*, 2018 ▸; Rosenthal *et al.*, 2014 ▸).

The electron-density distribution at the active site of *Pa*HSS molecule *D* did not indicate the binding of a PUT molecule. Inspection of molecule *F* showed distinct electron density at the active site, which might be caused by a PUT molecule similarly located as in molecules *A*, *C* and *E*. However, the density was not sufficient to accommodate a PUT molecule. While the longitudinal density was quite defined near Glu206 (the inner amino site), it did not extend all the way to the site surrounded by Trp225, His292 and the nicotinamide ring. This might indicate low occupancy of a potentially bound PUT molecule or increased flexibility of the PUT carbon chain at the latter site.

### 3.4. Structural comparison of *Pa*HSS and *Bv*HSS   

*Bv*HSS was previously reported to form dimers (Krossa *et al.*, 2016 ▸), which was equivalently confirmed by SEC-MALS in this study for the wild-type protein and all variants (the theoretical molecular weight of the wild-type and variant dimers was 106 kDa; the estimated molecular weight according to HPLC-MALS was 98–99 kDa; data not shown). While *Pa*HSS was found to be a monomer in solution (the theoretical molecular weight of the monomer and the estimated molecular weight according to HPLC-MALS were 52 kDa; data not shown), six monomers in the asymmetric unit form three crystalline dimers with significant interface areas (∼1330–1400 Å^2^, as determined by *PDBsum*; Laskowski *et al.*, 2018 ▸). Superimposition of the crystalline *Pa*HSS dimers onto the wild-type *Bv*HSS dimer (PDB entry 4tvb) revealed a highly similar quaternary structure of the *Bv*HSS and *Pa*HSS dimers, demonstrating a similar dimerization interface. However, the interface area of *Bv*HSS is larger (∼1700 Å^2^ according to *PDBsum*) than the interface areas of the crystalline *Pa*HSS dimers, which could explain the different oligomerization states of *Pa*HSS and *Bv*HSS in solution.

The overall structural similarity between *Pa*HSS (PDB entry 6y87 chain *A*) and *Bv*HSS (PDB entry 4tvb chain *B*; Krossa *et al.*, 2016 ▸) was examined by superimposition of both molecules and the subsequent calculation of a structure-based sequence alignment (Supplementary Fig. S5). This alignment shows a sequence identity of ∼44% and almost complete alignment of the structures. The few and short non-aligning gaps (C^α^ r.m.s.d. cutoff 5 Å) are exclusively caused by solvent-exposed parts of both structures, which are not involved in formation of the binding pocket (Supplementary Fig. S6). Superimpositions of the structures exclusively based on a single domain (either the Rossmann-like domain or the HSS-like domain) effectively showed the same alignment as observed for superimposition of the complete structures. In conclusion, *Pa*HSS and *Bv*HSS share equivalent intramolecular domain orientations.

In addition, a structure-based sequence alignment of *Pa*HSS (PDB entry 6y87 chain *A*) and *L. pneumophila* HSS (*Lp*HSS; PDB entry 2ph5) was performed (Supplementary Fig. S7). The resulting sequence identity is ∼41% and the structures align with high similarity and with an equivalent intramolecular domain orientation. Non-aligning parts (C^α^ r.m.s.d. cutoff 5 Å) are limited to a few solvent-exposed areas.

**Figure 6:**
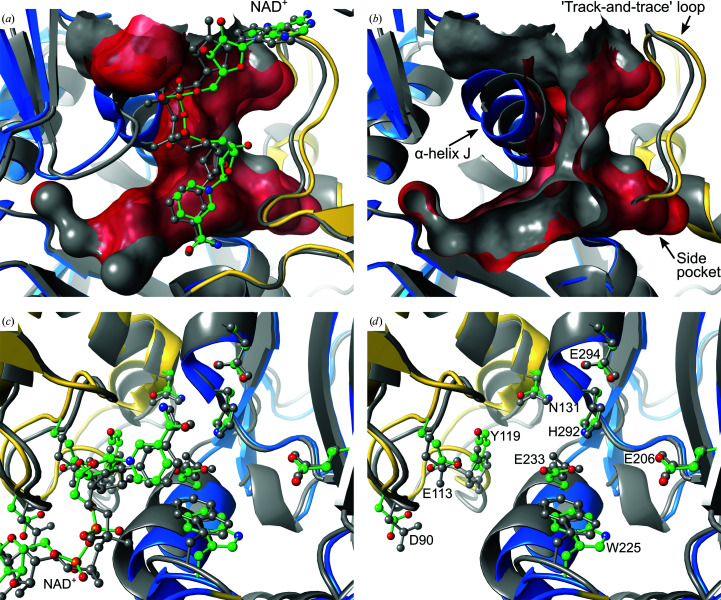
Structural comparison of *Pa*HSS and *Bv*HSS. Superimposition of *Pa*HSS (PDB entry 6y87 chain *A*, NAD(P)-binding Rossmann-like domain in yellow, HSS-like domain in blue) onto *Bv*HSS (PDB entry 4tvb chain *B*, gray; Krossa *et al.*, 2016 ▸). Protein models are depicted in cartoon representation with selected side chains and NAD^+^ molecules in ball-and-stick representation. (*a*, *b*) Depiction of the solvent-accessible binding pocket surfaces of *Pa*HSS (red, transparent) and *Bv*HSS (gray, opaque). In (*b*), the binding-pocket surfaces were clipped and the NAD^+^ molecules were hidden for clarity. (*c*, *d*) Depiction of selected side chains (numbered according to the *Pa*HSS sequence) in the superimposed binding pockets. In (*d*), the NAD^+^ molecules are hidden for clarity.

To further assess the similarity of the superimposed *Pa*HSS and *Bv*HSS structures, the architectures of their binding pockets were compared. Both binding pockets are ‘boot-shaped’ and are mainly defined by the respective ‘track-and-trace’ loop, α-helix J (*Pa*HSS residues 226–236; *Bv*HSS residues 230–240) and the NAD^+^ molecule (Figs. 6 ▸ *a* and 6 ▸ *b*). The NAD^+^ molecules similarly insert their nicotinamide rings into the active site of the binding pockets, albeit with a slight offset. In addition, the ‘track-and-trace’ loop and particularly α-helix J of *Pa*HSS are shifted in relation to *Bv*HSS, providing more space for the pocket entrance compared with the *Bv*HSS structure (Fig. 6 ▸ *b*).

This spatial effect was more closely inspected by measuring the dimensions of the binding-pocket entrances of *Pa*HSS and *Bv*HSS (Supplementary Fig. S8). The *Pa*HSS pocket entrance is shaped like a funnel, with a large opening (4.7 × 8.6 Å) which narrows further to a maximum of 3.5 Å. Mostly caused by the constricting α-helix J, *Bv*HSS has a smaller pore-like entrance (3.2 × 3.6 Å). Still, both entrance tunnels effectively impose similar spatial limitations for the access of compounds to the active sites. The ‘bottom’ of the binding pockets, which includes the active sites, provides comparable space in both structures.

To determine whether the reaction mechanism proposed for *Bv*HSS could also apply to *Pa*HSS, the locations and conformations of relevant residues within the binding pockets were compared between the superimposed structures. In general, these residues align with good agreement, albeit not to the same degree (Figs. 6 ▸ *c* and 6 ▸ *d*). Referring to the *Pa*HSS numbering, the side chains of Glu206, His292 and Glu294 align almost perfectly. Although *Pa*HSS residue Glu233 is part of the shifted α-helix J, its carboxyl group is still very similarly placed compared with the equivalent *Bv*HSS residue Glu237. While Glu113 and Tyr119 belong to the shifted ‘track-and-trace’ loop, Asp90 is part of another slightly displaced loop. These residues exhibit a certain offset but retain highly similar conformations and/or placement of functional groups. Trp225 belongs to a short loop region directly preceding α-helix J and is also affected by its shift in the *Pa*HSS structure. Apart from a small offset comparing the tryptophan residues of *Pa*HSS and *Bv*HSS, the indole rings are equally oriented and parallel to each other. Similarly, the NAD^+^ molecules are shifted with respect to one another but adopt highly similar conformations, including a coplanar alignment of their nicotinamide rings. In summary, the architecture of both binding pockets and especially the orientation of the relevant side chains are highly similar in both the *Pa*HSS and *Bv*HSS structures. The most significant deviation regarding catalysis might be the shifted tryptophan residues. Still, *Bv*HSS can be assumed to qualify as a mechanistic model for *Pa*HSS.

### 3.5. Cation–π interaction geometry in *Pa*HSS and *Bv*HSS   

**Figure 7:**
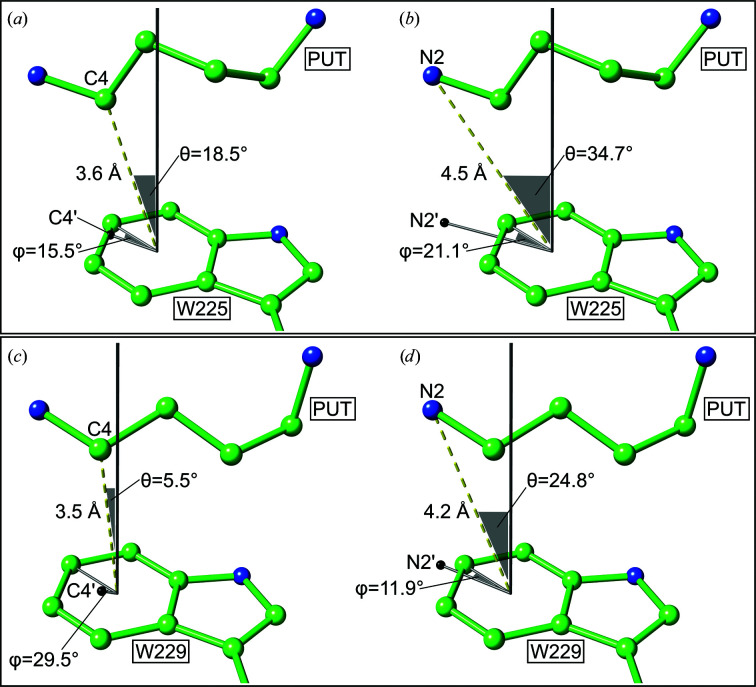
Geometry of cation–π interaction between PUT and tryptophan in *Pa*HSS and *Bv*HSS. Interaction geometry between PUT atoms C4 and N2 and the tryptophan benzene moiety in (*a*, *b*) *Pa*HSS (PDB entry 6y87 chain *E*) and (*c*, *d*) *Bv*HSS (PDB entry 4tvb chain *B*; Krossa *et al.*, 2016 ▸). Structures are shown in ball-and-stick representation, with distances as yellow dashed lines, angle legs as gray lines and angles as gray transparent triangles [not visible for φ in (*c*)]. The orthogonal projections of C4 and N2 onto the ring planes are shown as black spheres (C4′ and N2′). All angle legs originate from the centroid of the benzene moiety, including the dashed distance vectors (centroid to C4 and N2). The angle θ is spanned by the normal of the ring plane (gray, infinitely pointing upwards) and the C4 or N2 distance vector (yellow, dashed). The angle φ is between the vector pointing to C4′ or N2′ and the vector pointing to the CH2 ring carbon.

Cation–π interaction was previously proposed to be part of the reaction mechanism of *Bv*HSS (Krossa *et al.*, 2016 ▸). The negative electrostatic potential of Trp229 was suggested to interact with the positively charged amino group of substrates and intermediates, resulting in efficient positioning of the compounds in the active site. In addition, a more favorable cation–π interaction geometry was shown between the side chain of Trp229 and the C4 atom of the bound PUT molecule. Since the HSS reaction mechanism is based on nucleophilic attacks at C4 (Fig. 2 ▸, steps 3 and 6), cation–π interaction was suggested to stabilize a transient positive charge at C4, with a higher interaction energy compared with N2, to catalyze the reaction. In the following, cation–π interaction strengths are comparatively estimated for the *Pa*HSS structure (PDB entry 6y87 chain *E*) and the *Bv*HSS structure (PDB entry 4tvb chain *B*; Krossa *et al.*, 2016 ▸). Estimations are based on the inter­action geometry between the two PUT atoms N2 and C4 and the benzene moiety of the indole rings (Fig. 7 ▸). The PUT molecule in *Pa*HSS molecule *E* was chosen for the following comparison because it is most distinctly supported by all electron-density maps (Fig. 4 ▸) and provides the most favorable interaction geometry with regard to the C4 atom (an explanation is given below).

The geometry of cation–π interactions has already been statistically evaluated based on protein structures and correlated to binding energies by quantum-chemical calculations (Pinheiro *et al.*, 2017 ▸; Rapp *et al.*, 2014 ▸; Marshall *et al.*, 2009 ▸; Minoux & Chipot, 1999 ▸; Chipot *et al.*, 1996 ▸; Burley & Petsko, 1986 ▸). As rule of thumb, the binding energy of a cation–π interaction between the respective PUT atoms and the benzene moiety of tryptophan should increase with (i) decreasing centroid–cation distance, (ii) decreasing θ angle (with a maximum at 0°) and (iii) increasing φ angle (with a maximum at 30°; angles are explained in Fig. 7 ▸). However, the φ angle should play a minor role at the relatively small θ angles occurring in this study (Marshall *et al.*, 2009 ▸).

The binding of the substrate PUT is assumed to be stabilized at the ‘center amino site’ by cation–π interaction of its positively charged ‘outer’ ammonium moiety (atom N2). In *Pa*HSS molecule *E*, the geometry between PUT N2 and the benzene moiety exhibits an N2–centroid distance of 4.5 Å at a θ angle of 34.7° and a φ angle of 21.1°. The PUT N2 atoms are similarly located in the three *Pa*HSS molecules (*A*, *C* and *E*) and the interaction geometry between N2 and the tryptophan benzene moiety is effectively the same for the three bound PUT molecules. In *Bv*HSS, the distance amounts to 4.2 Å, with a θ angle of 24.8° and a φ angle of 11.9°. The cation–π interaction in *Bv*HSS should consistently be stronger due to a smaller cation–centroid distance and a smaller θ angle.

In addition, reaction intermediates with a transient positive charge at the C4 atom were proposed to be stabilized by cation–π interaction to facilitate a subsequent nucleophilic attack at C4 (Krossa *et al.*, 2016 ▸). These intermediates could include the protonated imine and the protonated 4-amino­butanal (Fig. 2 ▸, steps 3 and 5), in which the positive charge could transiently be shifted to the C4 atom, where it might subsequently be stabilized by cation–π interaction (Supplementary Fig. S2, steps 4 and 9, respectively). In *Pa*HSS molecule *E*, the PUT C4–centroid distance is 3.6 Å, with a θ angle of 18.5° and a φ angle of 15.5° (Fig. 7 ▸). The interaction geometries in the other *Pa*HSS molecules with bound PUT molecules are less favorable but still qualify as a cation–π interaction (*Pa*HSS molecule *A*, C4–centroid distance of 4.2 Å, θ angle of 26.4°; *Pa*HSS molecule *C*, C4–centroid distance of 4.8 Å, θ angle of 16.6°). In *Bv*HSS the distance is 3.5 Å, the θ angle is 5.5° and the φ angle is 29.5°, representing a near-optimal cation–π interaction geometry. While the C4–centroid distance in *Pa*HSS molecule *E* is very similar, the θ angle in particular indicates a less favorable geometry compared with *Bv*HSS. It should be noted that the PUT molecules detected in the crystal structures are not necessarily intermediates with a positive charge at C4 but are more probably regular substrates ‘waiting’ for further conversion. In particular, the inter­action geometry in *Pa*HSS should therefore be governed by cation–π interaction involving PUT N2 rather than C4. This could also explain the assumed multitude of conformations of PUT molecules, resulting in complicated and different tracing of the carbon chains in the respective *Pa*HSS molecules. Consistently, the stabilized amino groups (N2 atoms) of the bound PUT molecules are similarly located within the active sites (with equivalent cation–π interaction geometries), while the location of the presumedly unstabilized C4 atoms varies in relation to the tryptophan indole ring (with the most favorable cation–π interaction geometry in *Pa*HSS molecule *E*). However, a transient positive charge at PUT C4, occurring in the course of the catalytic reaction, would be stabilized with higher cation–π interaction energy according to the inter­action geometry than a positive charge at N2. In *Bv*HSS, the closer proximity of PUT C4 to the nicotinamide ring was interpreted as hydride transfer from PUT C4 to NAD^+^ C4N (Krossa *et al.*, 2016 ▸). This potential situation leaves more room for speculation regarding the oxidation state of PUT C4 and its resulting involvement in cation–π interactions.

In summary, the interaction geometry between the indole ring and both PUT N2 and PUT C4 in *Pa*HSS suggests significant cation–π interaction energies (Pinheiro *et al.*, 2017 ▸; Rapp *et al.*, 2014 ▸; Marshall *et al.*, 2009 ▸). However, the geometry in *Pa*HSS is less favorable and the interaction energies are thus assumed to be weaker than in *Bv*HSS. In conclusion, this inferior cation–π interaction geometry in *Pa*HSS might be the reason for its reduced activity compared with *Bv*HSS (see below).

### 3.6. Activity of *Pa*HSS, *Bv*HSS and *Bv*HSS variants   

To assess the effect of each single-residue mutation on *Bv*HSS catalysis and to compare the activities of wild-type *Bv*HSS and *Pa*HSS, the initial reaction velocities (*V*_0_) of the enzymes were determined under identical reaction conditions. *Bv*HSS has previously been reported to have a catalytic optimum at pH ∼8.8 (Tait, 1979 ▸; Ober *et al.*, 1996 ▸). To better reflect the physiological reaction conditions and considering the instability of *Pa*HSS at basic pH (thermal shift assays, not shown), assays were performed at neutral pH 7.4 and mostly at 310 K (Table 3 ▸, Supplementary Fig. S4).

As the only exception, comparison of wild-type *Bv*HSS and *Pa*HSS was performed at a reaction temperature of 298 K due to the inferior stability of *Pa*HSS at elevated temperatures (thermal shift assays, not shown). Wild-type *Bv*HSS was determined to exhibit a *V*_0_ of 0.34 ± 0.05 m*M* min^−1^ at 310 K and 0.108 ± 0.009 m*M* min^−1^ at 298 K. For *Pa*HSS, a *V*_0_ of 0.037 ± 0.004 m*M* min^−1^ at 298 K was determined, amounting to roughly 33% of the *Bv*HSS activity at 298 K. The reason for the lower activity of *Pa*HSS cannot be resolved at this point; the less favorable cation–π interaction geometry could be one explanation (see above). Another explanation could be the inferior stability of *Pa*HSS compared with *Bv*HSS in terms of pH and temperature, which might cause a certain fraction of the purified *Pa*HSS to be inactive/less active. However, protein aggregation was ruled out by the detection of a monodisperse *Pa*HSS elution peak corresponding to a monomer by HPLC-MALS analysis. Furthermore, the high structural similarity of the six *Pa*HSS molecules in the asymmetric unit of the crystal structure (PDB entry 6y87) did not indicate the presence of structurally impaired protein molecules. The temperature-dependence of *Bv*HSS is in line with previous studies, describing an increase in activity up to 318 K (Ober *et al.*, 1996 ▸). Activity assays were performed at a substrate concentration of 10 m*M* PUT dihydrochloride, which is sufficient for the oversaturation of wild-type *Bv*HSS (*Bv*HSS *K*_m_ = 0.26 m*M*; Böttcher *et al.*, 1994 ▸). The presented *V*_0_ value of *Bv*HSS at 310 K can accordingly be converted to a *k*_cat_ value of 1.5 s^−1^. This *k*_cat_ value lies between the previously reported *V*_max_ value of 22 nkat mg^−1^ (Böttcher *et al.*, 1994 ▸) (corresponding to a *k*_cat_ value of 1.2 s^−1^) and a specific activity of 83 nkat mg^−1^ (corresponding to a *k*_cat_ value of 4.4 s^−1^) (Tholl *et al.*, 1996 ▸). The reason for the distinct difference between the two reported values remains elusive. Also, the reported values were determined near the catalytic optimum at pH 8.5 and are thus expected to be higher than the value determined at pH 7.4 in this study. In any case, our assay provides comparable data and primarily serves for a relative catalytic comparison of the different *Bv*HSS variants in this study. More extensive activity assays will be required in the future to determine *K*_m_ and *k*_cat_ values for *Pa*HSS and the *Bv*HSS variants. However, our approach allowed the confirmation of significant effects of the single-residue mutations on HSP production. Considering the activity of wild-type *Bv*HSS at 310 K as a reference (0.34 m*M* min^−1^ = 100%), the D94N (61%) and D94K (52%) variants showed moderately reduced activity. The W229F (2%), E117Q (1.5%) and W229Y (1%) variants were still able to produce HSP from PUT, albeit with drastically decreased activity. The E117K, E210A, E210K, E210Q, W229A, W229E, W229H and W229K variants were completely inactive and did not show quantifiable production of HSP over 5 h (*V*_0_ < 9 × 10^−5^ m*M* min^−1^). These results are discussed below in the context of the structural evaluation of the variants.

### 3.7. Overall structures of *Bv*HSS variants   

All *Bv*HSS variants were solved in space group *P*22_1_2_1_ with similar unit-cell parameters and mostly refined to a resolution near or below 2 Å (Table 2 ▸; the maximum resolutions are mostly between 1.75 and 2.08 Å). The only exception was the structure of the *Bv*HSS variant W229E, which could only be refined to a maximum resolution of 2.76 Å. All *Bv*HSS structures were solved as dimers in the asymmetric unit, with each subunit containing an NAD^+^ molecule in the binding pocket. In addition, a PUT molecule was present at the active site of subunit *A* of *Bv*HSS variant W229A. Due to a lack of interpretable electron density, large parts of the ‘track-and-trace’ loops are missing in the *Bv*HSS variant E117Q (residues 123–127 are missing in chain *A* and residues 121–127 in chain *B*). This suggests high flexibility of the loop, as previously described for different *Bv*HSS variants (Krossa *et al.*, 2016 ▸).

The overall similarity of the *Bv*HSS variant structures was quantified by superimposition of the variant subunits onto wild-type *Bv*HSS subunit *B* (PDB entry 4plp) and subsequent calculation of the r.m.s.d. between all equivalent atoms (not only C^α^). The r.m.s.d. values ranged between 0.57 and 0.91 Å for the complete structures and between 1.48 and 1.64 Å (Supplementary Table S3) when only considering ‘track-and-trace’ loop residues 120–130 (not calculated for the E117Q variant due to missing residues). These r.m.s.d. values do not suggest significant perturbations of the *Bv*HSS variant structures. The increased structural flexibility and therefore deviation of the ‘track-and-trace’ loop was previously reported (Krossa *et al.*, 2016 ▸) and thus was expected. Furthermore, local inspection of the catalytically relevant residues in the binding pockets of the variants showed a high structural similarity to the wild-type residues where not stated otherwise in the following.

### 3.8. Requirement for negatively charged residues at the ‘ionic slide’ and the ‘inner amino site’   

**Figure 8:**
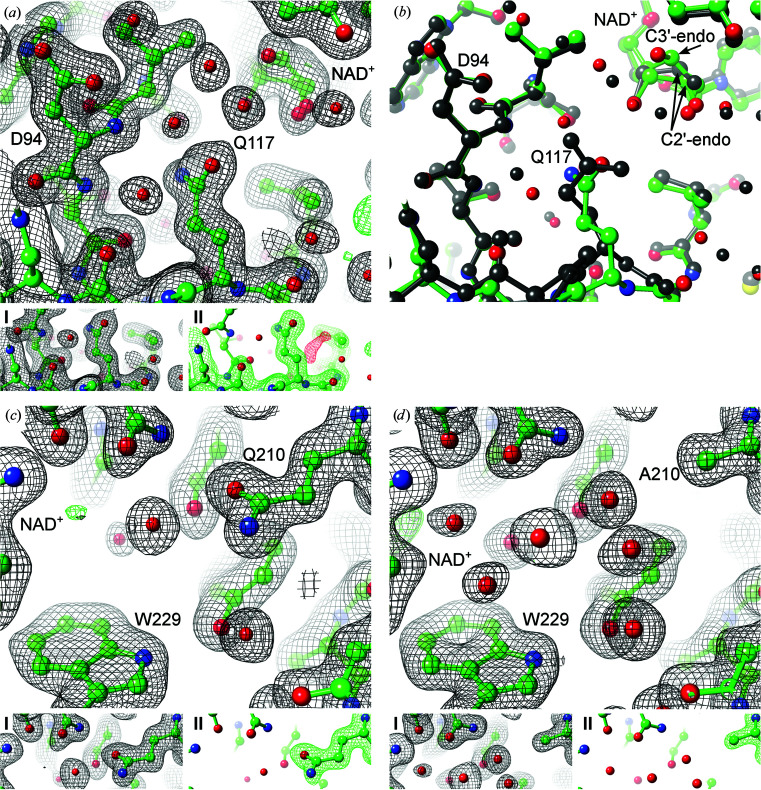
*Bv*HSS variants targeting the ‘ionic slide’ and the ‘inner amino site’. (*a*) *Bv*HSS variant E117Q (PDB entry 6s6g chain *B*), (*b*) superimposition of wild-type *Bv*HSS (PDB entry 4tvb chain *B*, gray) onto (*a*), (*c*) the E210Q variant (PDB entry 6s3x chain *A*) and (*d*) the E210A variant (PDB entry 6s49 chain *A*). Atomic models are depicted in ball-and-stick representation and electron-density maps as mesh. The large images (*a*, *c*, *d*) show the 2*mF*_o_ − *DF*_c_ maps (black, 1σ) and *mF*_o_ − *DF*_c_ maps (green/red, ±4σ) of the complete models. Models lacking the respective exchanged residue together with ten N-­terminally and ten C-terminally adjacent residues were used to calculate (I) 2*mF*_o_ − *DF*_c_ omit maps (black, 1σ) and (II) *mF*_o_ − *DF*_c_ omit maps (green/red, ±4σ) after refinement including simulated annealing.

For the attraction of protonated and thus positively charged amine substrates into the binding pocket of *Bv*HSS, Asp94 and Glu117 in particular were suggested to form an ‘ionic slide’. After reaching the active site, the ‘inner’ amino group of the reaction components is stabilized by Glu10. The importance of the ‘ionic slide’ was analyzed by exchange of the acidic residues for neutral and basic residues. The activity of these *Bv*HSS variants in relation to wild-type *Bv*HSS correlated to the negative electrostatic potential of the exchanged residue in the order D94N (61%) > D94K (52%) >>> E117Q (1.5%) > E117K (inactive). The deeper lying residue Glu117 consistently had far more influence on the activity than Asp94 at the pore opening. This effect might also be explained by an assumed lower dielectric constant, considering the protein environment of Glu117 compared with the more solvent-exposed environment of Asp94. Glu117 would consistently provide higher salt-bridge interaction energies for the attraction of positively charged substrates. The predominant role of Glu117 compared with Asp94 is also reflected by a sequence alignment of bacterial HSS orthologs, which revealed that only ∼40% of the enzymes contain an acidic residue at the position equivalent to Asp94 in *Bv*HSS (Shaw *et al.*, 2010 ▸). Only the *Bv*HSS variant E117Q (PDB entry 6s6g) provided sufficiently diffracting crystals for structure solution (Fig. 8 ▸ *a*).

While the architecture of the binding pocket in the variant closely resembles the wild-type pocket, a few minor structural deviations were detected. Large parts of the ‘track-and-trace’ loop are not traceable in the electron-density distribution (residues 123–127 and 121–127 are missing in subunits *A* and *B*, respectively), which is probably caused by the previously described flexibility (Krossa *et al.*, 2016 ▸) of this loop. Delocalization of the loops causes a shift of Gln117 compared with the wild-type residue Glu117 (Fig. 8 ▸ *b*). The shifted side chain of Gln117 is thereby moved away from the entrance tunnel and does not cause steric occlusion. In addition, the electron-density distribution of the NAD^+^ molecule was interpreted as alternative ribose conformers of the nicotinamide riboside moiety. While the ribose moiety in the wild-type structure is exclusively present as a C2′-*endo* conformer, both C2′-*endo* and C3′-*endo* conformers seem to be present in the *Bv*HSS variant E117Q. However, the extent of the above-described structural deviations is rather small and should not exert significant effects on the activity of the variant. Instead, the drastically reduced activity of the *Bv*HSS variant E117Q is assumed to be caused by the lack of negative charge at position 117. In line with this, the introduction of a basic residue in the *Bv*HSS variant E117K completely inactivated the enzyme. However, steric repulsion caused by the long lysine side chain or other structural disturbances cannot be ruled out without a structure of this variant. A simulated model of the *Bv*HSS variant E117K indicates that there are many possible conformations of Lys117. Therefore, the passage of educts and products through the entrance tunnel might be sterically hindered considering the length of the lysine side chain and the different possible conformations (see Supplementary Figs. S9*a* and S9*b* ).

Following a similar approach to probe the role of *Bv*HSS residue Glu210, the E210A, E210Q and E210K variants were examined and were found to be inactive. The crystal structures of the E210Q (PDB entry 6s3x; Fig. 8 ▸ *c*) and E210A (PDB entry 6s49; Fig. 8 ▸ *d*) variants were solved and showed clear contouring of the exchanged residues by electron density. Superimposition of the variant structures onto the wild-type structure revealed nearly identical locations of all residues in the binding pocket, including the wild-type and variant residues Glu210 and Gln210. These findings convincingly demonstrate the indispensable need for a negative charge at position 210 to stabilize the positively charged amino groups of the reaction component at the ‘inner amino site’.

### 3.9. Strong evidence for cation–π interaction between PUT and tryptophan at the active site   

**Figure 9:**
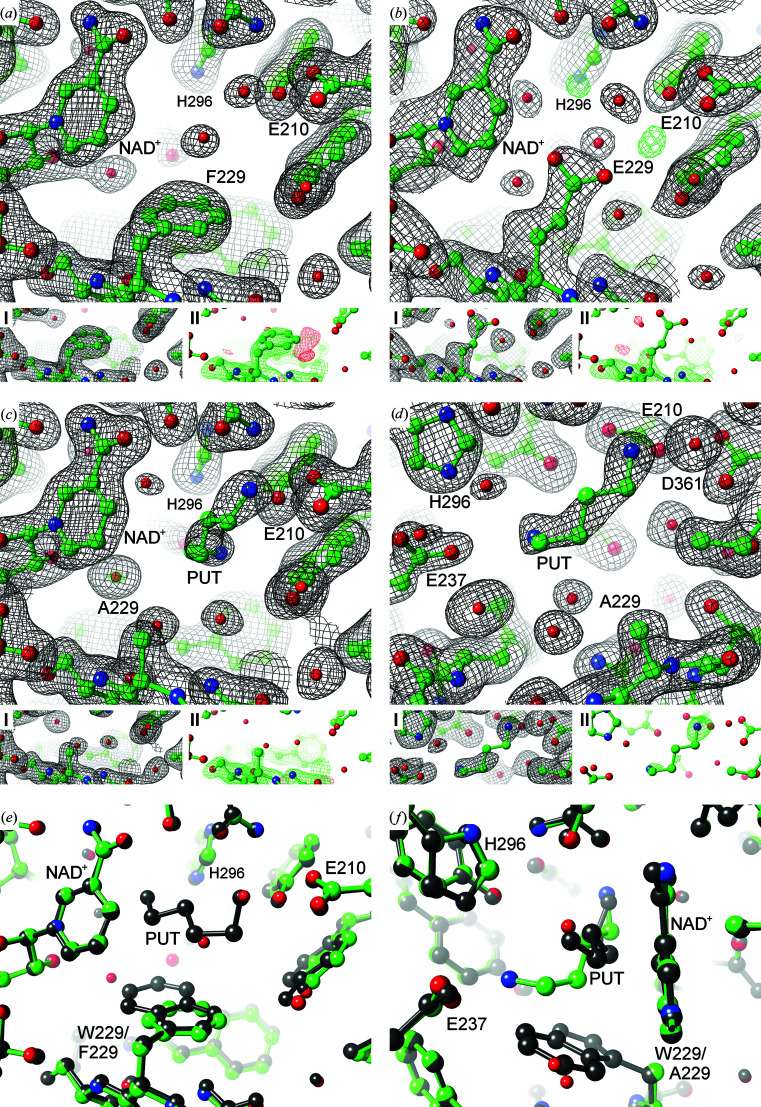
*Bv*HSS variants targeting the tryptophan at the active site. (*a*) *Bv*HSS variant W229F (PDB entry 6s4d chain *A*), (*b*) *Bv*HSS variant W229E (PDB entry 6sep chain *B*), (*c*, *d*) *Bv*HSS variant W229A (PDB entry 6s72 chain *A*) from different perspectives. (*e*, *f*) Superimposition of wild-type *Bv*HSS (PDB entry 4tvb chain *B*, gray; Krossa *et al.*, 2016 ▸) onto *Bv*HSS variant W229F (colored) (*e*) and *Bv*HSS variant W229A (colored) (*f*). Atomic models are depicted in ball-and-stick representation and electron-density maps as mesh. Large images (*a*–*d*) show the 2*mF*_o_ − *DF*_c_ maps (black, 1σ) and *mF*_o_ − *DF*_c_ maps (green/red, ±4σ) of the complete models. Models lacking the respective exchanged residue together with ten N-terminally and ten C-terminally adjacent residues [I and II in (*a*–*c*)] or lacking the PUT molecule [I and II in (*d*)] were used to calculate (I) 2*mF*_o_ − *DF*_c_ omit maps (black, 1σ) and (II) *mF*_o_ − *DF*_c_ omit maps (green/red, ±4σ) after refinement including simulated annealing.

The *Bv*HSS residue Trp229 has been proposed to play a central role in the reaction mechanism by stabilizing reaction components and intermediates by cation–π interaction. Until now, this assumption was exclusively based on evaluation of the interaction geometry between both the PUT and HSP molecules and the indole ring of Trp229 (Krossa *et al.*, 2016 ▸). To experimentally substantiate this interaction, the tryptophan residue was exchanged for residues with reduced aromatic character (W229F, W229Y and W229H), for both acidic (W229E) and basic (W229K) residues and for alanine (W229A). The W229E variant was generated to scrutinize the possible stabilization of positively charged reaction components by a salt bridge instead of a cation–π interaction. The W229K variant served as ‘negative control’, which should repel positively charged reaction components. Except for the W229F and W229Y variants, which retained very low activity (∼2% and 1% of the wild-type activity, respectively), all other variants were inactive. The crystal structures of the W229A, W229F and W229E variants could be solved and allowed several conclusions to be drawn in combination with the activity data. Apart from the exchanged residues, all residues in the binding pockets of these variants superimpose almost perfectly onto the wild-type residues without considerable deviations. In conclusion, the decreased or abolished activity should be caused by the exchange of Trp229 and not by structural perturbation of the binding pocket. In the W229F variant, the location of the Phe229 phenyl ring is distinctly defined by the electron-density distribution (Fig. 9 ▸ *a*).

Superimposition of the variant and wild-type structures shows coplanar alignment of the Phe229 phenyl ring and the wild-type Trp229 pyrrole moiety (Fig. 9 ▸ *e*). Consequently, the reduced activity of the variant is not caused by obstruction of the active site due to an inappropriately orientated phenyl ring. Without a bound PUT molecule in the active site of the structure, the exact location of the substrate cannot be determined. Catalytically unfavorable binding of the PUT molecule, also considering the smaller, less space-occupying phenyl ring, might therefore cause the decreased activity. However, the active site of *Bv*HSS is considerably narrow and both the W229F and W229Y variants provide bulky side chains to maintain the spatial dimensions. In the structure of the W229F variant, the side chain of the variant residue Phe229 sterically confines the active site to similar spatial dimensions as in wild-type *Bv*HSS. A substantially different binding of the PUT molecule in the narrow active site of the W229F variant therefore seems unlikely. Still, the activities of the W229F and W229Y variants are drastically reduced. In conclusion, we suggest the weaker cation–π interaction in the W229F and W229Y variants to be the reason for their reduced activities as explained in the following. Firstly, the Phe229 benzene moiety is shifted in relation to the Trp229 benzene moiety and therefore also in relation to the ‘center amino site’. The resulting cation–π interaction geometry in the W229F variant would consistently be more off-centered and therefore energetically less favored (Marshall *et al.*, 2009 ▸). Secondly, the phenyl ring (and similarly the phenol ring in the W229Y variant) exhibits less negative electrostatic potential and a smaller size of its π-electron system compared with the indole ring, resulting in a smaller cation–π interaction energy (Mecozzi *et al.*, 1996*b* ▸; Reddy & Sastry, 2005 ▸). The structure of the W229H variant could not be determined and correct positioning of the His229 side chain and the PUT molecule cannot be verified. Simulation of the possible side-chain orientations of His229 did not reveal steric hindrance or hydrogen-bond problems (Supplementary Figs. S9*e* and S9*f* ). However, notable cation–π interaction can be doubted even assuming optimal interaction geometry since the π system of the imidazole ring in the W229H variant provides even less negative electrostatic potential than the benzene and phenol rings (Mecozzi *et al.*, 1996*b* ▸; Reddy & Sastry, 2005 ▸). In line with these assumptions and considering the already low activity of the W229F and W229Y variants, the W229H variant was found to be completely inactive.

More evidence for cation–π interaction as a requirement for efficient catalysis might be inferred from the inactive variant W229A. Soaking the variant crystals with 0.2 *M* PUT for 5 min allowed the identification of a PUT molecule in the active site of subunit *A* (Figs. 9 ▸ *c* and 9 ▸ *d*). While stable binding of the substrate in the active site was possible in the variant, catalytic conversion was completely disrupted. Superimposition of this variant subunit onto the wild-type subunit also containing a PUT molecule (PDB entry 4tvb chain *B*; Krossa *et al.*, 2016 ▸) revealed different orientations of the PUT molecules (Fig. 9 ▸ *f*). The ‘inner’ amino group of both PUT molecules is similarly placed near the carboxyl group of Glu210. The ‘outer’ amino group in the W229A variant, however, is shifted towards or attracted by the carboxyl group of Glu237 (distance 3.3 Å) compared with the wild-type protein (distance 4.4 Å). In context of the oxidation of PUT C4 by NAD^+^ as part of the proposed reaction mechanism (Krossa *et al.*, 2016 ▸), the distance between PUT C4 and NAD^+^ C4N is 4.4 Å in the variant and 2.2 Å in the wild-type structure. An explanation for the inactivity of the W229A variant could therefore be the missing cation–π interaction, which prevents appropriate stabilization of the ‘outer’ amino group of PUT at the ‘center amino site’ near the nicotinamide ring. This could ultimately impede hydride transfer between PUT C4 and the nicotinamide ring as a prerequisite for imine formation (Fig. 2 ▸, steps 2–3). Another explanation would be the missing stabilization of a partial positive charge at PUT C4 to facilitate the nucleophilic attack of a water molecule (Supplementary Fig. S2, step 4), resulting in oxidative deamination (Krossa *et al.*, 2016 ▸). However, the lack of a bulky, confining side chain at position 229 broadens the active site and might thereby allow increased flexibility of the PUT molecule. This could also contribute to the inappropriate positioning of the PUT molecule in this variant, preventing further conversion of the substrate.

Regarding the structure of the inactive variant W229E, the location of the carboxyl group of Glu229 is probably not completely fixed. The carboxyl group is rotated between the two subunits of the variant and the omit maps reveal a lack of electron density clearly supporting the location of the carboxyl group (Fig. 9 ▸ *b*). A reduced occupancy of the carboxyl group at a defined location due to flexibility of the side chain or radiation damage affecting the carboxyl group should therefore be assumed. In any case, the side chain of Glu229 is able to protrude into the active site, as modeled in the variant structure, which would sterically interfere with substrate binding. The same principle might apply to the inactive variant W229K (for which no structure is available). Simulation of the W229K variant suggests a very diverse ensemble of side-chain rotamers, which interfere with the ‘inner amino site’ for positioning of the substrate (see Supplementary Fig. S9*g* ). In conclusion, the inactivity of both variants W229E and W229K is not necessarily caused by their inability to provide cation–π interaction but might rather result from steric issues or an enlarged space enabling increased flexibility of the substrate. Still, both variants are inactive as expected for a cation–π-dependent reaction mechanism. A future Asp229 variant might allow better evaluation of a salt bridge as potential compensation for a cation–π interaction without steric issues. However, the short side chain of an aspartate would require a distinct shift of PUT N2 from the active site towards Asp229 to form an energetically significant salt bridge (distance of ≤4 Å). Such a displacement would probably reduce or prevent efficient catalysis as proposed by the reaction mechanism.

In summary, the combination of activity assays and structural data convincingly suggests an indispensable catalytic need for strong cation–π interaction in *Bv*HSS and thus probably also in *Pa*HSS. The drastically decreased activity of the W229F and W229Y variants is particularly remarkable and emphasizes the need for (i) the more negative electrostatic potential and/or (ii) the more favorable interaction geometry provided by the indole ring. Exchange of Trp229 for non-aromatic residues generated inactive variants, which might be caused by the lack of cation–π interaction. However, other effects caused by the introduced residues might equivalently provide an explanation, including steric repulsion of the substrate or increased flexibility of the substrate in the enlarged space of the active site. As previously proposed by Krossa and coworkers, efficient catalysis might require the stabilization of both positively charged amino groups and/or carbons at the active site by cation–π interaction (Krossa *et al.*, 2016 ▸). In this context, the influence of cation–π interactions in catalysis for the stabilization of carbocations has been confirmed for aristolochene synthase (Faraldos *et al.*, 2011 ▸) and triterpenoid synthases (Hoshino, 2017 ▸; Chang *et al.*, 2013 ▸) by similar experimental approaches as in this study. The role of critical aromatic residues was also examined by site-directed mutagenesis and activity assays of the resulting variants, although structural data were limited to the wild-type enzyme or a homology model, respectively. To more precisely assess the requirement for cation–π interaction in bacterial HSS, finer modification of the interaction energy and thus of the resulting activity could possibly be achieved by the ‘fluorination strategy’ (Davis & Dougherty, 2015 ▸). This approach entails the replacement of relevant aromatic residues with fluorinated derivatives, which exhibit decreased cation–π interaction energies but very similar van der Waals radii compared with their native counterparts. Steric effects on catalysis, which cannot completely be ruled out in the case of exchange for native, sterically different amino acids, are negligible when using these derivatives. Also, the decline in activity would be expected to quantitatively correlate with the extent of fluorination of the introduced derivative. Simulation of *Bv*HSS variants with 5-fluorotryptophan or 6-fluorotryptophan introduced for Trp229 indicates equivalent orientations of the fluorinated indole rings and the wild-type indole ring without steric issues (Supplementary Fig. S10). These simulations suggest the feasibility of the corresponding experiments.

## 4. Conclusions   

Polyamines contribute to medically relevant processes in bacteria, including pathogens such as *P. aeruginosa*. The bacterial HSS is part of polyamine metabolism and exhibits high sequence conservation of catalytically relevant residues in different bacteria. Despite the potential medical significance of HSS in *P. aeruginosa* and other pathogenic bacteria, its role remains unexplored so far. Apart from an unpublished crystal structure of *L. pneumophila* HSS with moderate resolution (PDB entry 2ph5), structural as well as catalytic characterization is limited to the HSS from *B. viridis*. This study presents the crystal structure of *Pa*HSS in complex with its main substrate PUT. The architecture of the substrate-binding pocket and the conformation of catalytically relevant residues are highly similar to those of *Bv*HSS. Choosing *Bv*HSS as mechanistic model for *Pa*HSS (and for bacterial HSS in general), single-residue variants of *Bv*HSS have been characterized by structure determination and activity assays. The results emphasize the importance of attraction and binding of substrate amino groups by acidic residues via salt bridges at the entrance tunnel (‘ionic slide’) and at the ‘inner amino site’. Strong evidence for a catalytic dependence on cation–π interaction provided by the tryptophan residue at the active site is demonstrated. Exchange of this tryptophan for phenyl­alanine or tyrosine but not for histidine or non-aromatic residues can maintain catalysis, albeit with drastically reduced activity. These findings substantiate the previously proposed roles of the residues involved in the reaction mechanism of bacterial HSS and might provide guidelines for the design of HSS inhibitors.

## 5. Related literature   

The following reference is cited in the supporting information for this article: Webb & Sali (2016 ▸).

## Supplementary Material

PDB reference: homospermidine synthase from *Blastochloris viridis*, W229E variant, complex with NAD, 6sep

PDB reference: E210Q variant, complex with NAD, 6s3x

PDB reference: W229F variant, complex with NAD, 6s4d

PDB reference: E210A variant, complex with NAD, 6s49

PDB reference: E117Q variant, complex with NAD, 6s6g

PDB reference: W229A variant, complex with NAD and PUT, 6s72

PDB reference: from *Pseudomonas aeruginosa*, complex with NAD and PUT, 6y87

SEC-MALS, Supplementary Figures and Tables. DOI: 10.1107/S2059798321008937/ud5026sup1.pdf

## Figures and Tables

**Table 1:** Gradient composition of the reversed-phase HPLC Solvent *A*, 25 m*M* triethylamine (pH 4.8, adjusted with acetic acid); solvent *B*, 80%(*v*/*v*) acetonitrile; solvent *C*, methanol.

Time (min)	Solvent *A* (%)	Solvent *B* (%)	Solvent *C* (%)
0.0	100	0	0
1.0	78	22	0
15.0	78	22	0
27.0	55	39	6
27.5	53	33	14
34.0	20	10	70
37.0	0	100	0
57.0	0	100	0

**Table 2:** Data-collection and refinement statistics Values in parentheses are for the highest resolution shell.

Structure	*Pa*HSS	*Bv*HSS (E117Q)	*Bv*HSS (E210A)	*Bv*HSS (E210Q)	*Bv*HSS (W229A)	*Bv*HSS (W229E)	*Bv*HSS (W229F)
PDB code	6y87	6s6g	6s49	6s3x	6s72	6sep	6s4d
Data collection							
Resolution range (Å)	80.31–2.42 (2.51–2.42)	60.34–1.77 (1.83–1.77)	63.94–1.75 (1.81–1.75)	90.36–1.93 (2.00–1.93)	90.24–2.00 (2.07–2.00)	90.24–2.76 (2.86–2.76)	90.31–2.08 (2.15–2.08)
Wavelength (Å)	0.9763	1.0332	1.0332	0.9789	1.0332	1.0332	0.9763
Space group	*P*3_2_12	*P*22_1_2_1_	*P*22_1_2_1_	*P*22_1_2_1_	*P*22_1_2_1_	*P*22_1_2_1_	*P*22_1_2_1_
*a*, *b*, *c* (Å)	103.2, 103.2, 548.0	60.3, 110.6, 158.4	60.0, 109.8, 157.3	60.3, 110.1, 158.2	60.1, 110.0, 157.9	60.2, 109.9, 158.2	60.1, 110.0, 158.2
α, β, γ (°)	90, 90, 120	90, 90, 90	90, 90, 90	90, 90, 90	90, 90, 90	90, 90, 90	90, 90, 90
*R*_meas_	0.258 (1.312)	0.118 (0.720)	0.218 (0.769)	0.126 (0.810)	0.178 (0.759)	0.559 (1.927)	0.149 (0.905)
Total oscillation range (0.1° per image) (°)	100	150	180	150	160	280	160
〈*I*/σ(*I*)〉	6.4 (2.1)	8.9 (2.3)	6.2 (2.3)	9.5 (2.4)	7.0 (2.2)	5.4 (2.3)	9.1 (2.2)
Mosaicity (°)	0.078	0.048	0.083	0.094	0.065	0.173	0.094
CC_1/2_	0.979 (0.479)	0.975 (0.792)	0.984 (0.787)	0.973 (0.814)	0.988 (0.767)	0.774 (0.659)	0.994 (0.714)
Completeness (%)	99.0 (98.3)	99.3 (98.6)	99.9 (99.9)	97.7 (97.0)	99.9 (99.9)	99.9 (99.9)	99.3 (98.8)
Multiplicity	5.9 (5.9)	5.6 (5.6)	6.7 (6.7)	5.9 (5.9)	6.0 (6.1)	10.4 (10.6)	6.2 (6.2)
Refinement							
No. of unique reflections	127021 (12504)	103232 (10109)	105336 (10426)	78139 (7668)	71528 (7047)	27738 (2733)	63410 (6220)
*R*_work_/*R*_free_	0.191/0.231	0.160/0.188	0.151/0.181	0.152/0.184	0.155/0.199	0.169/0.216	0.158/0.195
CC*	0.995 (0.805)	0.994 (0.940)	0.996 (0.939)	0.993 (0.947)	0.997 (0.932)	0.934 (0.891)	0.999 (0.913)
CC_work_	0.942 (0.778)	0.966 (0.891)	0.955 (0.916)	0.969 (0.906)	0.964 (0.903)	0.947 (0.868)	0.967 (0.884)
CC_free_	0.913 (0.708)	0.948 (0.880)	0.957 (0.872)	0.962 (0.831)	0.937 (0.830)	0.862 (0.724)	0.957 (0.829)
No. of non-H atoms							
Protein	21615	7395	7468	7513	7597	7473	7539
Ligand/ion	282	199	116	116	94	88	127
Water	1242	877	1106	846	863	770	832
*B* factors (Å^2^)							
Protein	34.3	25.7	14.9	26.2	24.6	20.9	29.3
Ligand/ion	36.1	22.8	13.3	24.8	21.5	17.4	32.5
Water	34.5	36.3	26.5	35.5	33.7	25.1	36.1
R.m.s. deviations							
Bond lengths (Å)	0.003	0.005	0.011	0.011	0.009	0.003	0.010
Bond angles (°)	0.670	0.950	1.060	1.142	0.857	0.605	0.847
Ramachandran plot							
Most favored (%)	95.63	97.52	97.56	97.46	97.45	96.93	97.46
Allowed (%)	4.12	2.48	2.44	2.54	2.44	3.07	2.54
Outliers (%)	0.26	0	0	0	0.11	0	0
Matthews coefficient calculation†							
Nmol/asym	6	2	2	2	2	2	2
*V*_M_ (Å^3^ Da^−1^)	2.70	2.49	2.44	2.48	2.46	2.47	2.47
*P*(tot)	0.26	0.98	0.99	0.98	0.98	0.98	0.98

† Values derived from the Matthews coefficient calculator software within the *CCP*4 suite, where Nmol/asym is the number of protein molecules in the asymmetric unit, *V*_M_ is the Matthews coefficient and *P*(tot) is the probability across all resolution ranges.

**Table 3:** Catalytic activity of *Pa*HSS, *Bv*HSS and *Bv*HSS variants The time-resolved production of HSP was quantified after the addition of 10 m*M* PUT dihydrochloride. Initial reaction velocities were determined from the linear fit to the initial data points of the progression curves.

Protein	*T* (K)	Initial velocity *V*_0_ (m*M* min^−1^)
*Bv*HSS wild type	310	0.34 ± 0.05
*Bv*HSS D94N	310	0.20 ± 0.01
*Bv*HSS D94K	310	0.17 ± 0.02
*Bv*HSS W229F	310	0.0067 ± 0.0006
*Bv*HSS E117Q	310	0.005 ± 0.001
*Bv*HSS W229Y	310	0.0038 ± 0.0003
*Bv*HSS wild type	298	0.108 ± 0.009
*Pa*HSS wild type	298	0.037 ± 0.004
*Bv*HSS E117K	310	<9 × 10^−5^ (inactive)
*Bv*HSS E210A	310	<9 × 10^−5^ (inactive)
*Bv*HSS E210K	310	<9 × 10^−5^ (inactive)
*Bv*HSS E210Q	310	<9 × 10^−5^ (inactive)
*Bv*HSS W229A	310	<9 × 10^−5^ (inactive)
*Bv*HSS W229E	310	<9 × 10^−5^ (inactive)
*Bv*HSS W229H	310	<9 × 10^−5^ (inactive)
*Bv*HSS W229K	310	<9 × 10^−5^ (inactive)
