# Supplementary material for: Structural and catalytic characterization of Blastochloris viridis and Pseudomonas aeruginosa homospermidine synthases supports the essential role of cation–π interaction
Source: Acta Crystallogr D Struct Biol. 2021 Sep 23;77(Pt 10):1317–35. doi: 10.1107/S2059798321008937 (PMC8489232; doi:10.1107/S2059798321008937)
Supplement: Supplementary file 1 [file d-77-01317-sup1.pdf]

Acta Cryst D STRUCTURAL BIOLOGY

Volume 77 (2021)

Supporting information for article:

Structural and catalytic characterization of *Blastochloris viridis* and *Pseudomonas aeruginosa* homospermidine synthases supports the essential role of cation–π interaction

F. Helfrich and Axel J. Scheidig

S1. SEC-MALS

The oligomerization state of proteins was determined by SEC-MALS at an HPLC system (Agilent 1100 series (Waldbronn, Germany), vacuum degasser (S 8515, Schambeck SFD GmbH, Bad Honnef, Germany), quaternary pump (G1311A), autosampler (G2258), multi-wavelength detector (G1365B), refractive index detector (G1362A), three-angle light scattering detector (miniDAWN TREOS, Wyatt Technology, Dernbach, Germany)) equipped with a WTC-050N5 column (4.6x300 mm, 500 Å pore size, 5 µm particle size, Wyatt Technology) protected by a WTC-050N5G guard column (Wyatt Technology). Protein samples were isocratically separated in PBS (pH 7.0) at 0.4 mL/min. The M_W of the analyzed samples was determined by the software Astra 5.3.4.10 (refractive index n_{water}=1.331, $dn/dc_{protein}$=0.185 mL/g, second virial coefficient A_2=0, Model=Zimm, fit degree=1). Conventional calibration of the column was done with a protein standard mix (15-600 kDa, Sigma-Aldrich, Darmstadt, Germany).

Table S1 Primers used for the cloning of *Bv*HSS variants.

Mutated nucleotides in reference to the wildtype *Bv*HSS sequence are colored red. The corresponding codon is underlined.

**Name of primer**	**Sequence (5' → 3')**
*Bv*HSS D94K fw	TCTCGGTCAAAACCTCATCGCT
*Bv*HSS D94K rv	GATGAGGTTTTGACCGAGAGGT
*Bv*HSS D94N fw	CCTCTCGGTCAATACCTCATCGCTC
*Bv*HSS D94N rv	GAGCGATGAGGTATTGACCGAGAGG
*Bv*HSS E117K fw	CGTGGTCAAACCGTGGCTTG
*Bv*HSS E117K rv	CCACGGTTTGACCACGGTGT
*Bv*HSS E117Q fw	ACACCGTGGTCCAGCCGTGGCTT
*Bv*HSS E117Q rv	AAGCCACGGCTGGACCACGGTGT
*Bv*HSS E210A fw	CATCGCCGCGCGCGACA
*Bv*HSS E210A rv	TGTCGCGCGCGGCGATGT
*Bv*HSS E210K fw	ACATCGCCAAGCGCGACA
BvHSS E210K rv	TCGCGCTTGGCGATGTG
*Bv*HSS E210Q fw	ACATCGCCCAACGCGACA
*Bv*HSS E210Q rv	TCGCGTTGGGCGATGTG
*Bv*HSS W229A fw	AACACCGCGTCGGTCGAG
*Bv*HSS W229A rv	ACCGACGCGGTGTTGACG
*Bv*HSS W229E fw	TTCGTCAACACCGAATCGGTCGAG
*Bv*HSS W229E rv	CTCGACCGATTCGGTGTTGACGAA
*Bv*HSS W229F fw	CGTCAACACCTTTTCGGTCGAGGG
*Bv*HSS W229F rv	CCCTCGACCGAAAAGGTGTTGACG
*Bv*HSS W229H fw	TCAACACCCATTCGGTCGAGGG
*Bv*HSS W229H rv	TCGACCGAATGGGTGTTGACGA
*Bv*HSS W229K fw	GTTCGTCAACACCAAGTCGGTCGA
*Bv*HSS W229K rv	TCGACCGACTTGGTGTTGACGAAC
*Bv*HSS W229Y fw	TCAACACCTATTCGGTCGAGGG
*Bv*HSS W229Y rv	TCGACCGAATAGGTGTTGACGA
T7 promoter	TAATACGACTCACTATAGGG
T7 terminator	GCTAGTTATTGCTCAGCGG

Table S2 Superimposition of *Pa*HSS molecule A onto the other *Pa*HSS molecules of the asymmetric unit.

The *Pa*HSS molecules (PDB ID 6Y87) were superimposed using the "super" algorithm over 5 cycles in PyMOL (only protein atoms, alternate locations A and NAD^+ atoms, no hydrogens). RMSD values were calculated with the command "rms_cur". For each RMSD value, the number of superimposed atom pairs is given in parenthesis. These numbers differ due to missing residues in the structures of the respective *Pa*HSS molecules (missing residues in molecule B: 1-3; C: 1-5 and 449-458; D: 1-4 and 449-457; E: 1-3 and 451-456; F: 1-3 and 450-457).

***Pa*HSS chain A compared to**	**RMSD (atom pairs)**
*Pa*HSS chain B	0.791 Å (3675)
*Pa*HSS chain C	0.828 Å (3597)
*Pa*HSS chain D	0.807 Å (3605)
*Pa*HSS chain E	0.827 Å (3640)
*Pa*HSS chain F	0.729 Å (3619)

Table S3 Superimposition of wildtype *Bv*HSS subunit B onto *Bv*HSS variant subunits.

Wildtype *Bv*HSS subunit B (PDB ID 4TVB chain B (Krossa *et al.*, 2016)) was superimposed onto the subunits listed in the first column using the "super" algorithm over 5 cycles in PyMOL (only protein atoms, alternate locations A and NAD^+ atoms, no hydrogens). RMSD values were calculated with the command "rms_cur" for the complete subunits including the NAD^+ molecules (column 2) and for the "track-and-trace" loop residues 120-130 (column 3). For each RMSD value, the number of superimposed atom pairs is given in parenthesis. Wildtype *Bv*HSS subunit B contains 3752 atoms (only protein atoms, alternate locations A and NAD^+ atoms, no hydrogens). Lower numbers of superimposed atoms are due to the introduced mutations, missing residue 477 in variants E117Q, E210A, E210Q, W229E and missing "track-and-trace" loop residues in variant E117Q.

***Bv*HSS (PDB ID 4TVB chain B) compared to**	**RMSD of complete subunits (atom pairs)**	**RMSD of residues 120-130 (atom pairs)**
*Bv*HSS (PDB ID 4TVB chain A)	0.709 Å (3752)	0.566 Å (93)
*Bv*HSS variant E117Q (PDB ID 6S6G chain A)	0.913 Å (3688)	high flexibility, missing residues 123-127
*Bv*HSS variant E117Q (PDB ID 6S6G chain B)	0.760 Å (3673)	high flexibility, missing residues 121-127
*Bv*HSS variant E210A (PDB ID 6S49 chain A)	0.600 Å (3734)	1.542 Å (93)
*Bv*HSS variant E210A (PDB ID 6S49 chain B)	0.731 Å (3734)	1.483 Å (93)
*Bv*HSS variant E210Q (PDB ID 6S3X chain A)	0.612 Å (3734)	1.582 Å (93)
*Bv*HSS variant E210Q (PDB ID 6S3X chain B)	0.748 Å (3734)	1.638 Å (93)
*Bv*HSS variant W229A (PDB ID 6S72 chain A)	0.598 Å (3737)	1.488 Å (93)
*Bv*HSS variant W229A (PDB ID 6S72 chain B)	0.796 Å (3737)	1.494 Å (93)
*Bv*HSS variant W229E (PDB ID 6SEP chain A)	0.669 Å (3729)	1.564 Å (93)
*Bv*HSS variant W229E (PDB ID 6SEP chain B)	0.736 Å (3729)	1.583 Å (93)
*Bv*HSS variant W229F (PDB ID 6S4D chain A)	0.569 Å (3737)	1.509 Å (93)
*Bv*HSS variant W229F (PDB ID 6S4D chain B)	0.804 Å (3737)	1.640 Å (93)

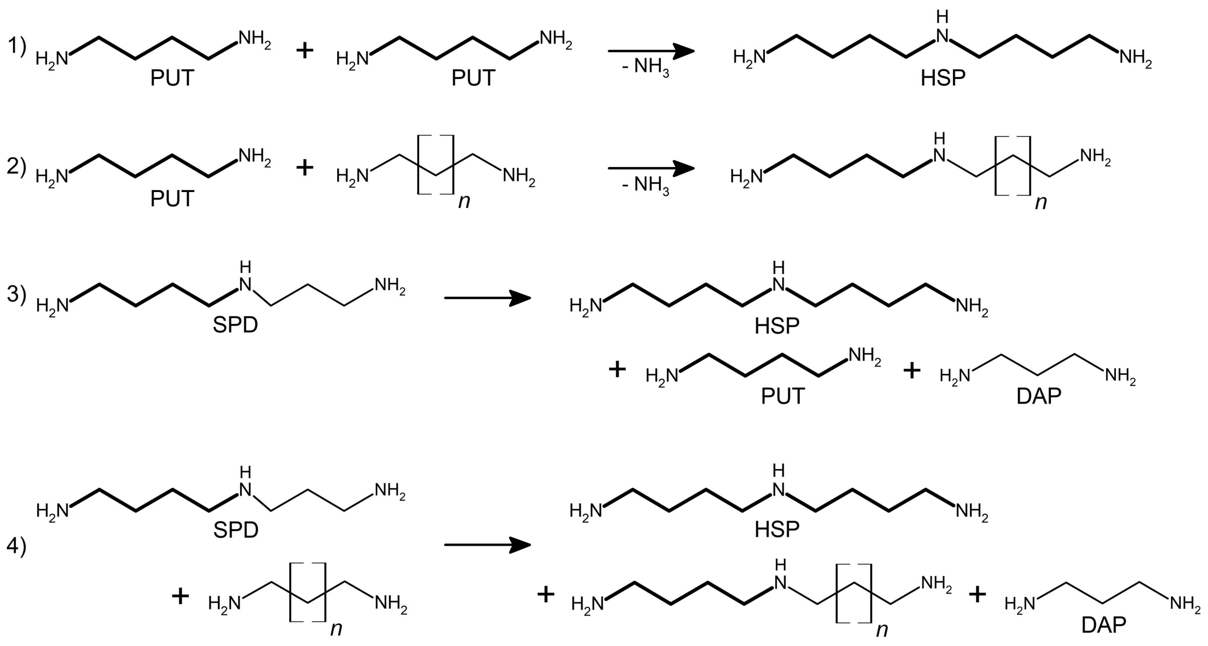

Figure S1 Reactions catalyzed by HSS (Ober *et al.*, 1996; Böttcher *et al.*, 1994; Tait, 1979). Bonds originating from PUT are depicted thicker. **1)** Main reaction, two PUT molecules are converted into HSP; **2)** PUT and a second diamine (n=1 to 5) are converted into a corresponding triamine; **3)** SPD as sole substrate is converted into HSP, PUT and DAP; **4)** SPD and a diamine (n=3 to 4) are converted into HSP, a corresponding aminobutyl-containing triamine and DAP. DAP=1,3-diaminopropane, HSP=*sym*-homospermidine, PUT=putrescine, SPD=spermidine. Reactions 3) and 4) are not balanced since they remain to be fully elucidated regarding the presence and further conversion of predicted intermediates (Ober *et al.*, 1996).

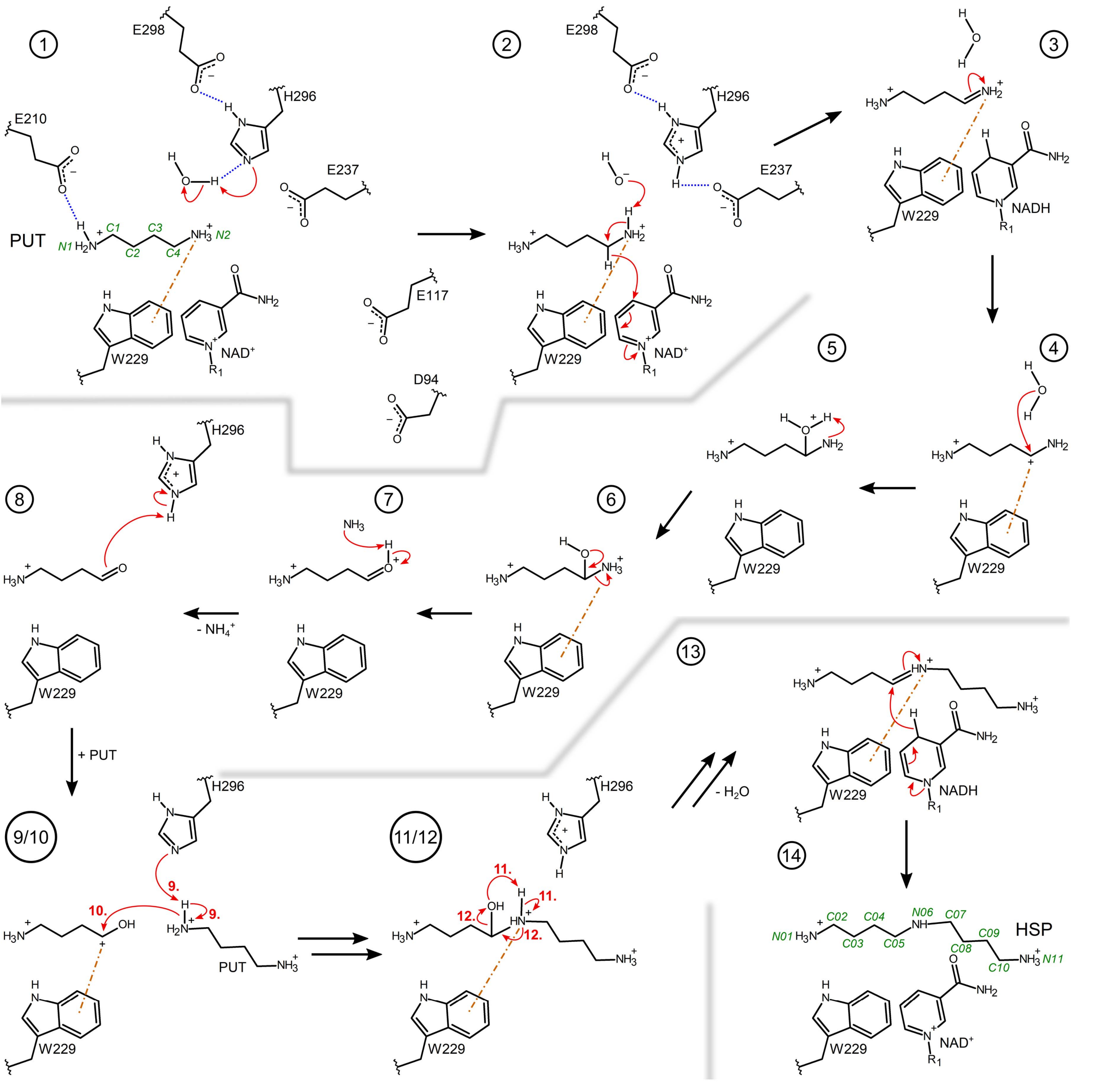

Figure S2 Proposed reaction steps of the conversion of PUT to HSP by the bacterial HSS. Relevant residues, NAD(H), PUT, HSP and intermediates are shown as two-dimensional structure representations. Hydrogen bonds are depicted as blue dotted lines, delocalized electrons as dashed lines, cation-π interactions as orange dash-dotted lines and electron transfers as red arrows. Atom numbering is given for PUT and HSP in green. For simplicity, steps 9 and 10 as well as steps 11 and 12 are shown in combined depictions with correspondingly labeled electron transfers. Additional intervening reaction steps compared to **Fig. 2** are shown in this figure based on a more detailed, previously proposed mechanism of reaction steps (Krossa *et al.*, 2016).

CCATGGATTTTAGCATTAATCCGCCGCAGCGTATTGTTTTTGTTGGTCTGGGTACCATTGCGCAGTC
ATTTCTGCCGCTGCTGAGCAAAGTTCATGATCTGTCTACCCTGGAAATTTATGCCATTGATCCGAA
AACCCCGCCGCTGATTGAATATTTTGCCAATTCTTTTGGCCTGAAATTTATTAATAGCGCCATTGAT
CAGATTAATTATCGCGATATTCTGGTGCCGATTCTGGGCGAAGGTACCGTGCTGATTAATCTGTCA
ACCGATGTGAGCAGCCTGGCCCTGATTGAACTGTGCCGCTCTGCTGGTGCGCTGTATCTGGATACC
TGCATTGAACCGTGGAAAGGTGGTTATGATGATCCGACCATTCCGCTGCATAAACGTACCAATTAT
CATCTGCGCGAACAGATGCTGAGCCTGAAAAAACGCCTGGGTAGTGGCGTGACCGCTCTGGTTGC
CCACGGTGCTAATCCGGGTCTGGTTTCACATTTTGTGAAACGTGCCCTGCTGGATCTGGCAGAAGA
AATTCTGGGTGATTGCAAAAAACCGTCTAATAAAGAACAGTGGGCCATTCTGTCTCAGCGTCTGGG
CGTGAAAGTTATTCATGTTGCAGAATATGATTCACAGATTTCTCAGAAATCACGCGAACGCGGTGA
ATTTGTGAATACCTGGAGCGTGCATGGCTTTATTTCAGAAAGCCAGCAGCCGGCGGAACTGGGTTG
GGGTTCTCATGAACGTTCACTGCCGACCGATGCTAGTATGCATACCGATGGCTGTGGTGCGGCAAT
TTATATTGAAAAACCGGGTGCCAGCGTTCGTGTGAAAACCTGGACCCCGTTTAATGGCCCGTCTCT
GGGTTATCTGGTTACCCATCATGAAGCCATTTCAATTGCGGATTTTCTGACCCTGCGCACCGCGGA
TGAAACCTATCGCCCGACCGTTCATTATGCTTATCGTCCGAGTGATGAAGCTATTCTGTCTGTTCAT
GAATGGTTTGGCAATGATTGTATGACCCCGGAAAAAACCAAAGTTCTGCGTCCGGGCGATATTCTG
AGCGGTTCTGATTATCTGGGCGTGCTGCTGATGGGCCATGAAAAATCAAGCTATTGGTATGGCAGT
ATTCTGAGCATTGAAAAAGCTAAAGAACTGGCGACCCTGAATACCGCTACCACCCTGCAGGTGGC
AGCAGGCGTTCTGTCAGGCTATCTGTGGATTCTGTCTCATCCGTCAGCAGGTATTATTGAAGCAGA
AGATATGGATCATGAAGTTGCACTGAGTTATATTAGTCAGTATCTGGGTGAACTGAAAGGTGTTTA
TAGTGATTGGAATCCGACCAAAAATAATCCGGGCACCTTTAGTGCGATTGATAGTGATAGTCCGTG
GCTGTTTAGTAATTTTGTGCTGTAATAACTCGAG

Figure S3 DNA sequence of *Pa*HSS including cloning sites and stop codons. The *Pa*HSS amino acid sequence (UniProtKB Q6X2Y9) was back-translated into an *E. coli* K12 codon-optimized DNA sequence (black). Addition of 5'-CC (green) and TAATAACTCGAG-3' to the termini of the DNA sequence provided 5’-NcoI (underlined) and 3’-XhoI (blue, underlined) restriction sites and two stop codons (red).

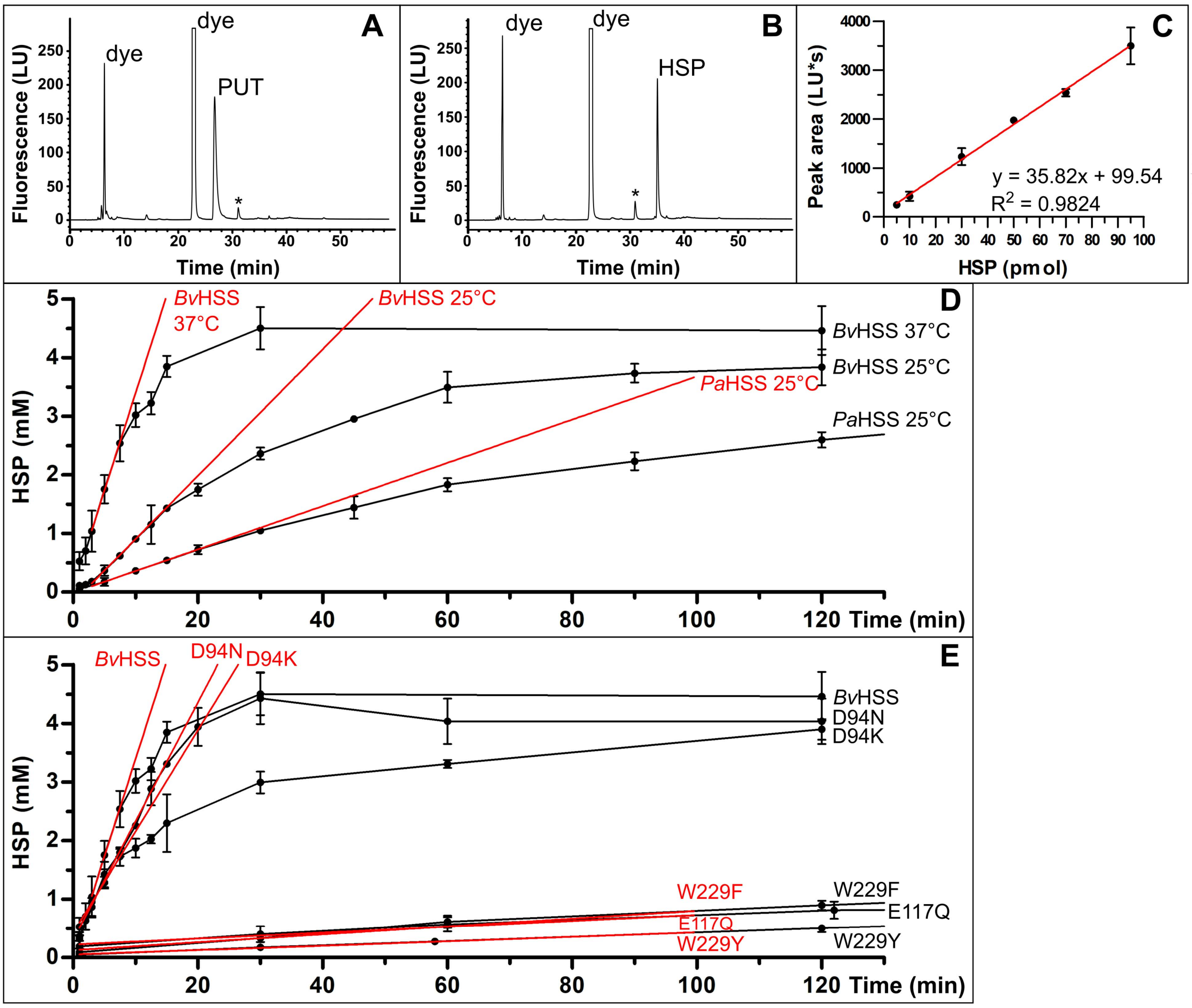

Figure S4 Activity assays of *Pa*HSS, *Bv*HSS and *Bv*HSS variants. (**A**) and (**B**) C-18 chromatograms of 200 pmol labeled PUT (A) and 95 pmol labeled HSP (B), * unidentified peak. (**C**) HSP standard curve, fluorescence peak areas of different amounts of labeled HSP as dots (mean of n=3, bars: ± standard deviation) and the linear regression as red line. (**D**) and (**E**) Progression curves (dots: mean of n=3, bars: ± standard deviation bars, connecting black lines) of the conversion of 10 mM PUT to HSP, catalyzed by the enzymes and reaction temperatures as indicated. The slope of the linear fit (red) to the initial, linear part of each progression curve (considering data of at least three time points), provides the initial velocity. (E) Wildtype *Bv*HSS and *Bv*HSS variants at 310 K.

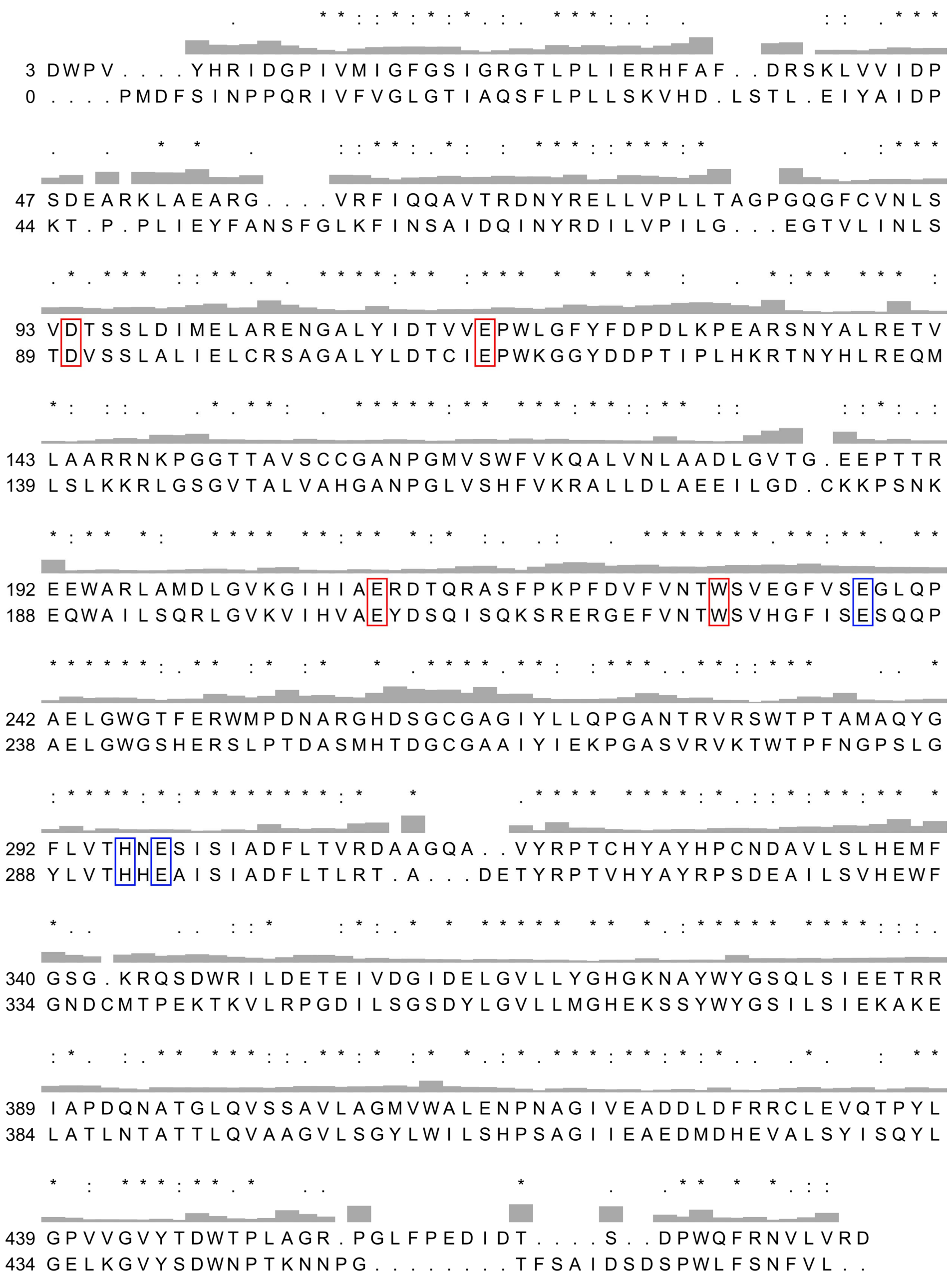

Figure S5 Structure-based sequence alignment of *Bv*HSS and *Pa*HSS. Superimposition of *Bv*HSS (upper sequence, PDB ID 4TVB chain B) onto *Pa*HSS (lower sequence, PDB ID 6Y87 chain A) using the UCSF Chimera 1.14 “MatchMaker” tool, followed by structure-based sequence alignment (Cα RMSD cutoff 5 Å). The numbering corresponds to the respective amino acid sequences. Residue number “0” of *Pa*HSS corresponds to the leftover of the expression tag. Conservation is depicted as Clustal symbols according to the PAM250 matrix (* = identical, : = high similarity, . = low similarity) and RMSD values between complete residues are given as histogram (RMSD cutoff 5 Å). Residues exchanged in the respective *Bv*HSS variants (D94, E117, E210 and W229) and corresponding *Pa*HSS residues are highlighted with red boxes, the triad composed of *Pa*HSS/*Bv*HSS residues E233/E237, H292/H296 and E294/E298 with blue ones.

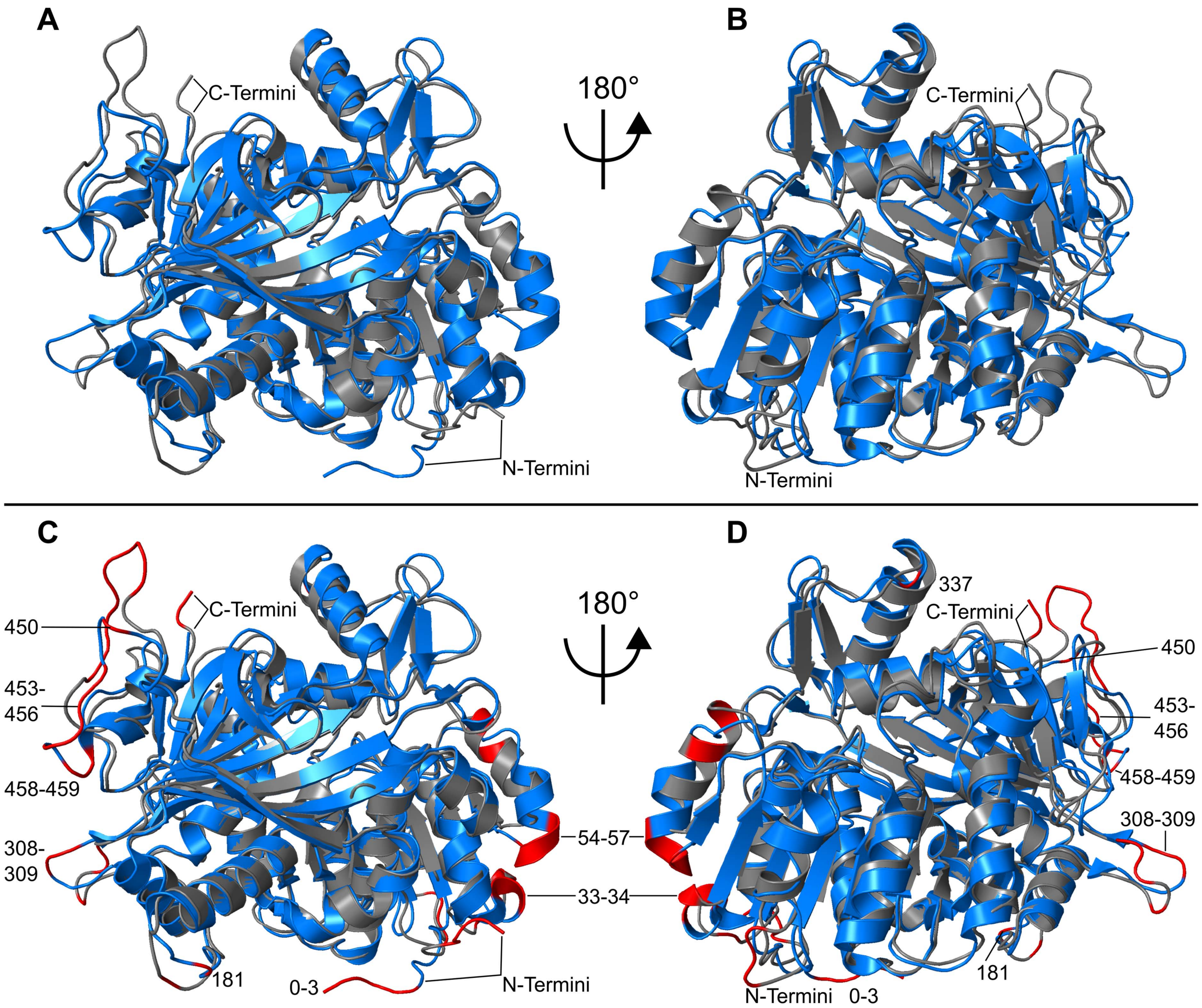

Figure S6 Structural superimposition of *Pa*HSS and *Bv*HSS. (**A**) and (**B**) Cartoon representation of *Pa*HSS (blue, PDB ID 6Y87 chain A) and *Bv*HSS (grey, PDB ID 4TVB chain B). N- and C-termini are labeled. (**C**) and (**D**) correspond to (A) and (B), respectively. Parts of both structures with RMSD of > 5Å from each other are additionally colored in red. Complete residues were considered for calculation of RMSD as explained in **Fig. S5** and the red parts of the structures correspond to the non-aligning sequences in **Fig. S5**. The numbers of the *Pa*HSS residues with RMSD of > 5Å and the N and C-termini are labeled.

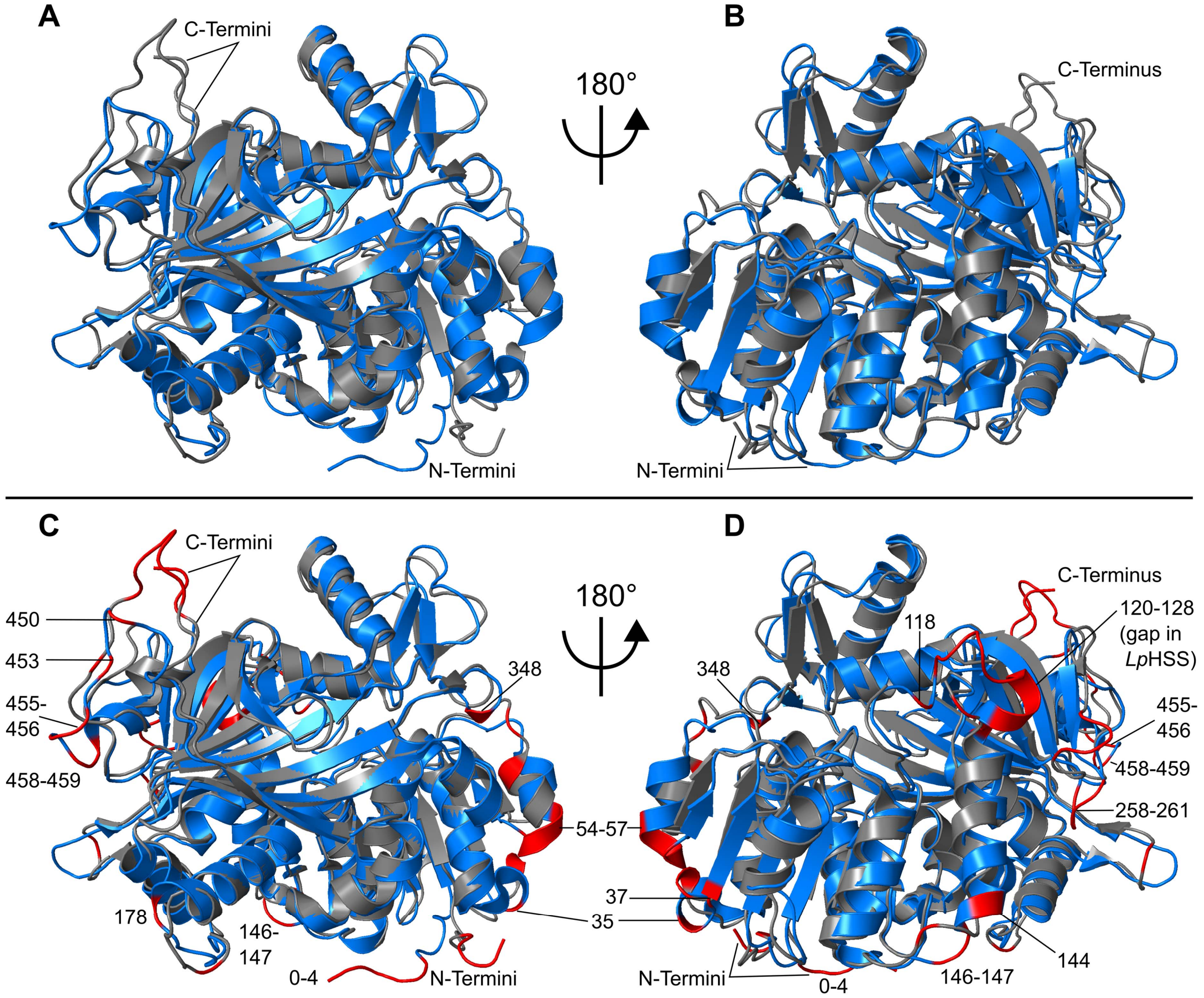

Figure S7 Structural superimposition of *Pa*HSS and *Legionella pneumophila* (*Lp*HSS). (**A**) and (**B**) Cartoon representation of *Pa*HSS (blue, PDB ID 6Y87 chain A) and *Lp*HSS (grey, PDB ID 2PH5). N- and C-termini are labeled. (**C**) and (**D**) correspond to (A) and (B), respectively. Parts of both structures with RMSD of > 5Å from each other are additionally colored in red. Superimposition was performed using the UCSF Chimera 1.14 "MatchMaker" tool followed by structure-based sequence alignment considering complete residues for calculation of RMSD. The numbers of the non-aligning *Pa*HSS residues with RMSD of > 5Å and the N- and C-termini of both structures are labeled. Residues 120-128 in the *Pa*HSS structure mostly "misalign" due to a gap in the *Lp*HSS structure (*Lp*HSS residues 121-130 missing).

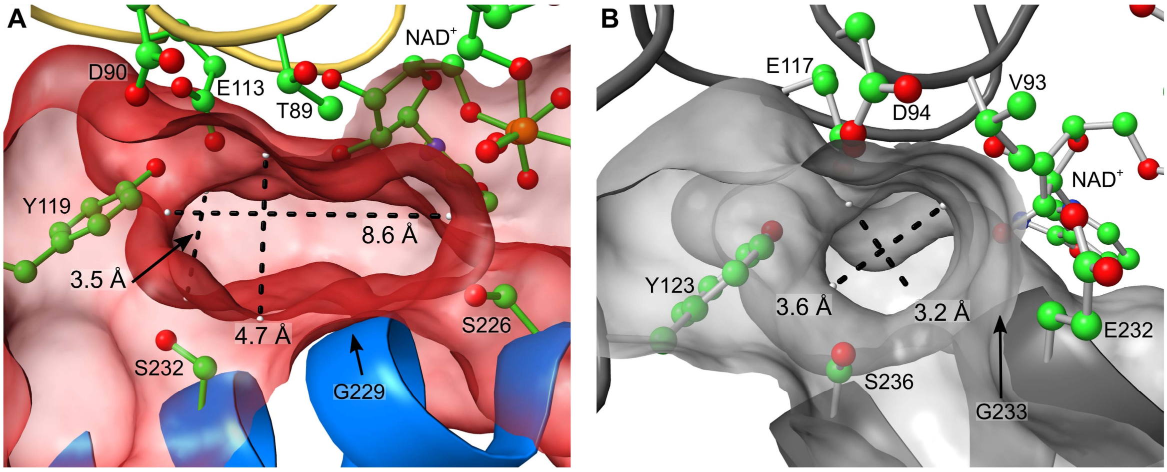

Figure S8 Dimensions of the binding pocket entrances of *Pa*HSS and *Bv*HSS. (**A**) *Pa*HSS (PDB ID 6Y87 chain A), (**B**) *Bv*HSS (PDB ID 4TVB chain B). Protein models are depicted as cartoon representation, selected side chains lining the upper entrance tunnel and NAD^+ molecules as ball-and-stick. The surfaces of the solvent-accessible binding pockets were rendered transparently. Distances were measured with the program PyMOL and illustrated by dashed lines connecting white spheres at the surface of the binding pockets. Distances were measured in the same plane within each structure except for the distance of 3.5 Å in (A), which was measured further below the entrance. The implicit locations of the residues G229 and G233 are indicated.

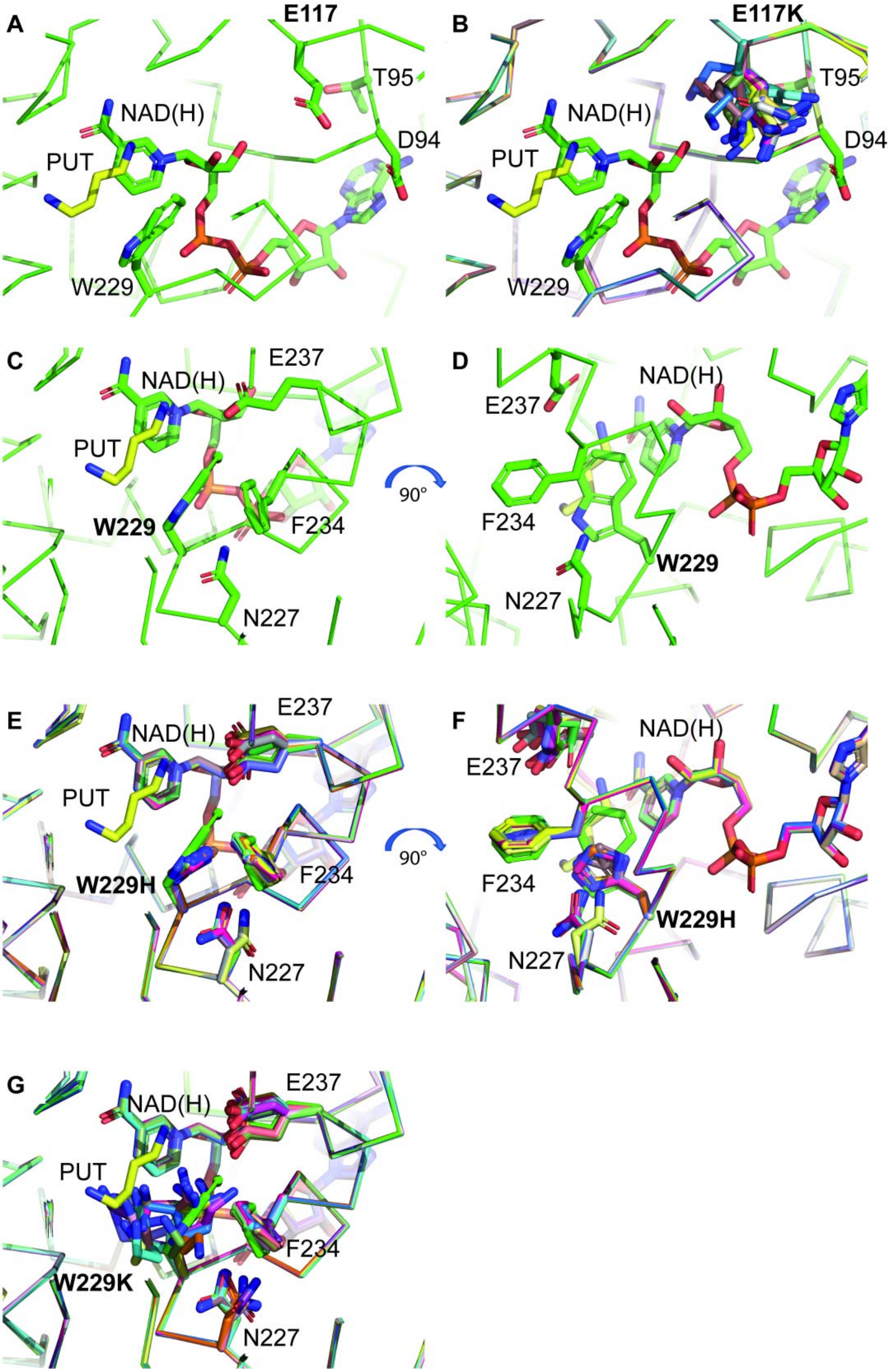
A
E117
T95
NAD(H)
D94
PUT
W229
B
E117K
T95
NAD(H)
D94
PUT
W229
C
E237
NAD(H)
PUT
W229
F234
N227
90°
D
NAD(H)
E237
F234
W229
N227
E
E237
NAD(H)
PUT
W229H
F234
N227
90°
F
NAD(H)
E237
F234
W229H
N227
G
E237
NAD(H)
PUT
F234
W229K
N227

Figure S9 Simulation of side chain conformations of *Bv*HSS variants which could not be crystallized for crystal structure determination. The program MODELLER (Webb & Sali, 2016) was used to calculate 25 independent structures of the respective *Bv*HSS variant using the chain B of wildtype *Bv*HSS for introduction of the respective single residue mutations (PDB ID 4TVB (Krossa *et al.*, 2016)). The NAD^+ cofactor was included in the simulation while the bound PUT molecule was neglected. The program was used with default values and no special restraints were set. By this procedure, only those conformations that do not enter into spatial overlaps with neighboring residues were selected by the simulation. All pictures were generated using the program PyMOL (Schrödinger). Structures are mainly depicted in wire representation and selected residues, the NAD^+ molecule and the PUT molecule in stick representation. Carbon atoms of the wildtype structure and the NAD^+ cofactor are colored green, carbon atoms of the PUT molecule are yellow and carbon atoms of variant structures are differently colored. (**A**) Wildtype *Bv*HSS structure (PDB ID 4TVB chain B). (**B**) Display of the conformational flexibility of residue K117 in the *Bv*HSS variant E117K superimposed onto the wildtype structure. (**C**/**D**) Display of the wildtype *Bv*HSS structure in different orientations. (**E/F)** Display of the side chain orientations of the residues N227, H229, F234 and E237 in the *Bv*HSS variant W229H as deduced by the simulations superimposed onto the wildtype structure. The views of panel (**E**) and (**F**) are identical to the views of panel (**C**) and (**D**), respectively. (**G**) Display of the conformational flexibility of the residues N227, K229, F234 and E237 of the *Bv*HSS variant W229K superimposed onto the wildtype structure. The view of panel is identical to the view of panel (**C**).

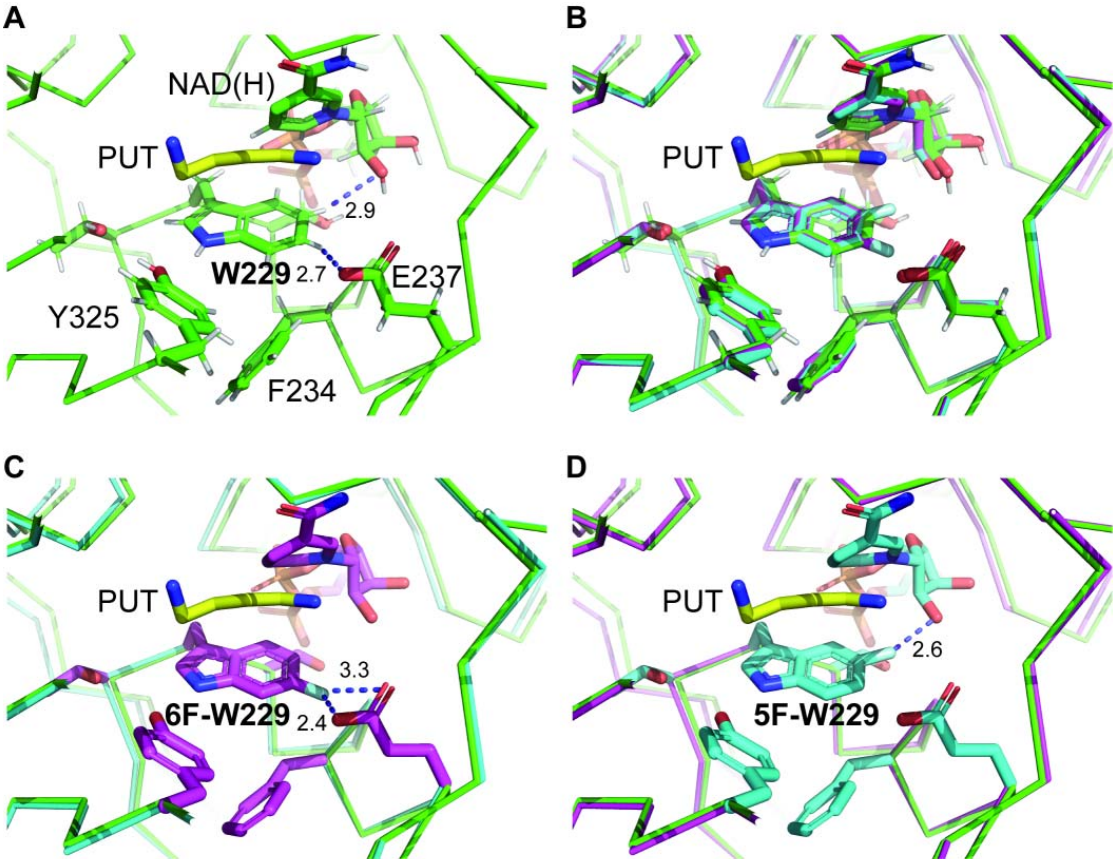

Figure S10 Simulation of fluorinated tryptophan side chains in the active site of *Bv*HSS. The program REFMAC5 within the CCP4 program suit was used to calculate the *Bv*HSS structure with W229 replaced by either 5-fluoro-tryptophan or 6-fluoro-tryptophan. The PDB entry 4TVB (Krossa *et al.*, 2016) was used as the initial structure for introduction of the fluorinated tryptophan residues. The structural parameters for the fluorinated tryptophan residues are based on the REFMAC dictionary (e.g. monomer FTR for 6-fluoro-tryptophan). All pictures were generated using the program PyMOL (Schrödinger). Structures are mainly depicted in wire representation and selected residues, the NAD^+ molecule and the PUT molecule in stick representation. Carbon atoms of the wildtype structure and the NAD^+ cofactor are colored green, carbon atoms of the PUT molecule are yellow and carbon atoms of structures with fluorinated tryptophan residues are colored cyan or magenta. (**A**) Display of the wildtype *Bv*HSS structure. The distance between the hydrogen atom H-5 of residue W229 and the oxygen atom of the nicotinamide riboside 2’-OH group as well as the distance of the hydrogen atom H-6 of residue W229 to the carboxyl group oxygen of E237 are given in angstrom. (**B**) Display of the wildtype *Bv*HSS structure superimposed onto the simulated structures of *Bv*HSS with 5-fluoro-tryptophan and 6-fluoro-tryptophan, respectively. (**C**) Display of the simulated *Bv*HSS (6F-W229) structure. The distances of the fluorine atom at the tryptophan position 6 to the side chain of E237 is given in angstrom. (**D**) Display of the simulated *Bv*HSS (5F-W229) structure. The distance of the fluorine atom at the tryptophan position 5 to the oxygen atom of the nicotinamide riboside 2’-OH group is given in angstrom.

References

Böttcher, F., Ober, D. & Hartmann, T. (1994). *Can. J. Chem.* **72**, 80–85.

Krossa, S., Faust, A., Ober, D. & Scheidig, A. J. (2016). *Sci. Rep.* **6**, 19501.

Ober, D., Tholl, D., Martin, W. & Hartmann, T. (1996). *J. Gen. Appl. Microbiol.* **42**, 411–419.

Tait, G. H. (1979). *Biochem. Soc. Trans.* **7**, 199–201.

Webb, B. & Sali, A. (2016). *Current protocols in bioinformatics* **54**, 5.6.1-5.6.37.
